# Inhibition of adenylyl cyclase by GTPase-deficient Gα_i_ is mechanistically different from that mediated by receptor-activated Gα_i_

**DOI:** 10.1186/s12964-024-01572-3

**Published:** 2024-04-05

**Authors:** Yin Kwan Chung, Ho Yung Chan, Tung Yeung Lee, Yung Hou Wong

**Affiliations:** 1 Division of Life Science and Biotechnology Research Institute, Hong Kong University of Science and Technology, Hong Kong, China; 2 State Key Laboratory of Molecular Neuroscience, and the Molecular Neuroscience Center, Hong Kong University of Science and Technology, Clear Water Bay, Kowloon, Hong Kong, China; 3 Rudolf Schönheimer Institute of Biochemistry, Division of General Biochemistry, Medical Faculty, Leipzig University, Johannisallee 30, 04103 Leipzig, Germany

**Keywords:** AC inhibition, G protein-coupled receptor, Receptor activation, Effector recognition domain, Constitutively active mutants

## Abstract

Signal transduction through G protein-coupled receptors (GPCRs) has been a major focus in cell biology for decades. Numerous disorders are associated with GPCRs that utilize G_i_ proteins to inhibit adenylyl cyclase (AC) as well as regulate other effectors. Several early studies have successfully defined the AC-interacting domains of several members of Gα_i_ by measuring the loss of activity upon homologous replacements of putative regions of constitutive active Gα_i_ mutants. However, whether such findings can indeed be translated into the context of a receptor-activated Gα_i_ have not been rigorously verified. To address this issue, an array of known and new chimeric mutations was introduced into GTPase-deficient Q204L (QL) and R178C (RC) mutants of Gα_i1_, followed by examinations on their ability to inhibit AC. Surprisingly, most chimeras failed to abolish the constitutive activity brought on by the QL mutation, while some were able to eliminate the inhibitory activity of RC mutants. Receptor-mediated inhibition of AC was similarly observed in the same chimeric constructs harbouring the pertussis toxin (PTX)-resistant C351I mutation. Moreover, RC-bearing loss-of-function chimeras appeared to be hyper-deactivated by endogenous RGS protein. Molecular docking revealed a potential interaction between AC and the α3/β5 loop of Gα_i1_. Subsequent cAMP assays support a cooperative action of the α3/β5 loop, the α4 helix, and the α4/β6 loop in mediating AC inhibition by Gα_i1-i3_. Our results unveiled a notable functional divergence between constitutively active mutants and receptor-activated Gα_i1_ to inhibit AC, and identified a previously unknown AC-interacting domain of Gα_i_ subunits. These results collectively provide valuable insights on the mechanism of AC inhibition in the cellular environment.

**Supplementary Information:**

The online version contains supplementary material available at 10.1186/s12964-024-01572-3.

## Introduction

G protein-coupled receptors (GPCRs) constitute a major class of cell surface receptors with characteristic 7-transmembrane helices. A plethora of diverse cellular activities that ranges from transcription [1], secretion [2], to cell migration [3] and proliferation [4] are orchestrated by GPCRs and their associated G proteins. Many GPCRs that signal through members of the G_i_ family have tremendous therapeutic value because they serve as key detectors and regulators in various physiological systems. For instance, G_i_-coupled opioid receptors are the primary targets for opiate analgesics and their prolonged activation will inevitably lead to opiate tolerance and physical dependence [5]. Likewise, altered expression or function of G_i_-coupled receptors are associated with various psychiatric disorders [6] including the serotonin 5-HT_1B_ receptor in depression [7], dopamine D_2_ receptor in bipolar disorder [8], and α_2A_-adrenergic receptor in schizophrenia [9]. Dysregulated G_i_-coupled receptor signaling can also result in other chronic ailments such as inflammatory bowel disease [10], Alzheimer’s disease [11], and heart failure [12].

Although many G_i_-coupled receptors are capable of regulating multiple signaling pathways, they invariably inhibit adenylyl cyclase (AC) via both pertussis toxin (PTX)-sensitive and PTX-insensitive members of the Gα_i_ subfamily (namely, Gα_i1-3_ and Gα_z_) [13, 14]. The molecular basis by which these Gα_i_ subunits inhibit AC, however, has not been completely elucidated. Distinct preference for specific Gα_i_ subunits has been reported for several GPCRs [15, 16], but there is little indication on whether such preferences have a determining effect on agonist-induced inhibition of AC. It remains to be established if Gα_i1-3_ and Gα_z_ utilize the same structural domains to interact with AC. Early chimeric studies have utilized GTPase-deficient mutants (mutation of the conserved Arg or Gln in the GTPase domain into Cys or Leu, respectively; henceforth referred to as RC or QL mutants) to map the effector-binding domains of Gα_i2_ and Gα_z_ [17–19], because replacement of the critical effector recognition domains on the mutants with homologous regions from other Gα subunits would abolish their constitutive inhibitory action on AC. These studies have provided valuable clues on the general location of the AC recognition domain in spite of a lack of Gα_i_-AC structural data. The putative AC interaction domain of Gα_i2_ was mapped across the switch II, α3 helix, α3/β5 loop and the α4/β6 loop [17, 18], with the latter structure in Gα_z_ similarly implicated in effector recognition [19]. While the putative AC-interacting regions identified in these Gα_i_ subunits are in line with the known effector domains of other Gα subunits such as Gα_s_ [20], the precise molecular determinants for AC inhibition by Gα_i_ remain elusive. A recent structural study on Gα_t1_ and Gα_s_ have further implicated the involvement of the αG/α4 loop in effector recognition [21]. A phenylalanine residue (F283) on the αG/α4 loop of Gα_t1_ is seemingly essential for effector activation, and mutation of the cognate residue (F312) on Gα_s_ also abolishes the activity of Gα_s_QL [21].

The interchangeable use of RC and QL mutants in various experiments, including the early mapping studies [17–19], assumes that both GTPase-deficient mutants behave similarly. Yet, several reports have hinted at potential functional differences between the two mutations. For instance, an I25A mutation on Gα_q_ was shown to eliminate the constitutive stimulation of phospholipase Cβ (PLCβ) by the RC, but not the QL, mutant [22]. Another study on the oncogenic potentials of constitutively active Gα_i_ mutants observed that only mutation on Gln204, but not Arg178, of Gα_i1_ suppressed cAMP formation in NIH/3T3 fibroblasts [23]. Moreover, GTP hydrolysis of Gα_i1_RC, but not Gα_i1_QL, was accelerated by RGS4 (a regulator of G protein signaling) when assayed with purified recombinant proteins [24]. These provided clues that QL and RC mutations may have intrinsic differences which have been overlooked in earlier studies, even though they both impede GTP hydrolysis and result in constitutive activation of the Gα subunits. Fundamentally, the extent to which the two constitutively active mutants resemble a receptor-activated Gα subunit, which is more physiologically relevant, have not been carefully examined.

Given that activated Gα_i_ members are known to interact with proteins other than AC, such as regulators of G protein signaling (RGS) proteins [25] and G protein regulated inducer of neurite outgrowth 1 [26], it is pertinent to identify residues that specify distinct signaling or regulatory outcome. Hence, in the present study, a series of Gα_i1_ chimeras with the putative effector-interacting domains replaced by homologous regions of Gα_t1_ or Gα_q_ were constructed with or without a GTPase-deficient mutation (QL or RC), and the chimeras were tested for their ability to abolish the constitutive activity. The reasons of choosing Gα_i1_ as a model to examine QL and RC mutations are multifold. Firstly, functional difference between Gα_i1_QL and Gα_i1_RC have been reported [23, 24]. Secondly, Gα_i1/t1_ chimeras were extensively used for deciphering effector-binding regions of transducin [21, 27, 28]. Mapping studies on Gα_i2_, which shares > 90% homology with Gα_i1_, also provide clues on putative AC-interacting domains of Gα_i1_ [17, 18]. Thus, the activities of Gα_i1_ chimeras harboring the QL or RC mutation can be readily tested to infer the functionality of the two mutants. Our results clearly suggest that there exist functional differences between Gα_i1_QL and Gα_i1_RC, and that the receptor-driven active conformation of Gα_i1_ is functionally more efficient than GTPase-deficient mutants of Gα_i1_ in suppressing the activity of AC. Moreover, we identified α3/β5 loop as an additional region generally utilized by Gα_i1-3_ for AC inhibition. These findings shed light on the mechanism of Gα_i_ to elicit its effect in a biological context upon receptor activation.

## Results

### *Design and expression of Gα*_*i1*_* chimeras*

**Fig. 1:**
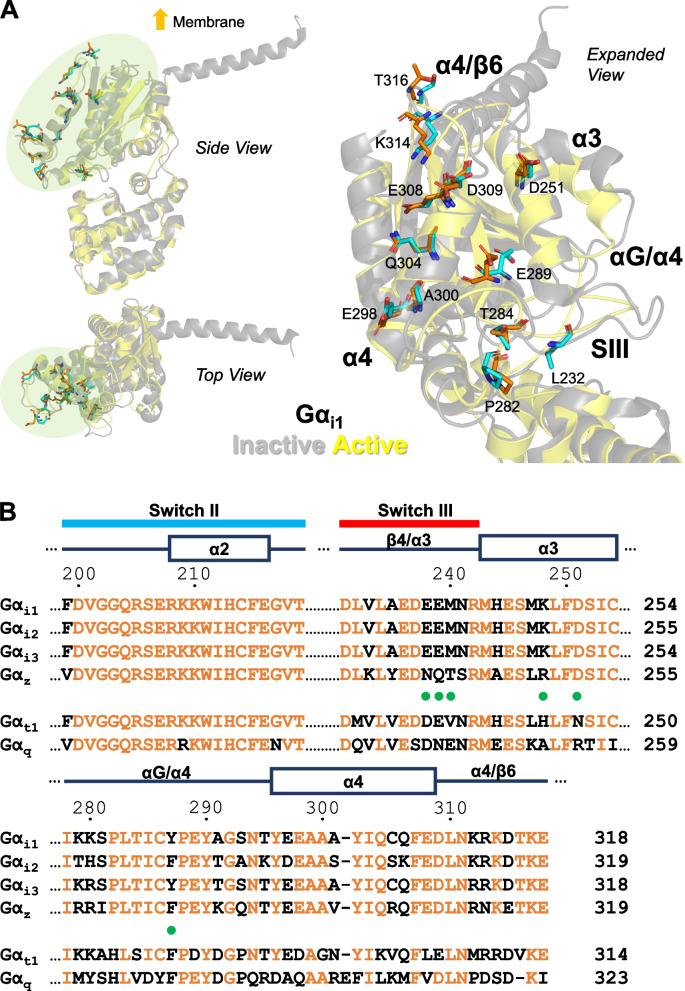
Putative AC-interacting domains of Gα_i1_. **A** The 3-dimensional structures of the GTPase domains of inactive (gray, PDB code: 1GP2) and active Gα_i1_ (yellow, PDB code: 1GFI) are overlaid and displayed as side, top and expanded views. The putative AC-interacting domains are marked in pale green (for side and top views) or labeled in the expanded view. Residues that are strictly conserved in AC-inhibiting Gα_i1-3_ and Gα_z_ are shown as cyan (inactive) or orange (active) sticks in the expanded view. **B** Amino acid sequence alignment of the putative AC-inhibiting regions between Gα_i1-3_, Gα_z_, and the homologous regions of Gα_t1_ and Gα_q_. Conserved residues are indicated in orange. Residues subjected to point mutations in the chimeric studies are annotated with green dots

Although the AC-interacting domains of Gα_i1_ have not yet been elucidated, designing an effector-deficient Gα_i1_ chimera to test for abolishment of QL/RC-driven constitutive activities was made feasible by previous mutagenesis and structural studies of other Gα subunits (such as Gα_i2_), because Gα_i1-3_ show remarkably high homology (~ 90% with respect to Gα_i1_) [29]. Moreover, several regions identified in previous mapping studies [17–19, 27, 30] correspond to potential effector binding sites in the crystal structures of Gα_t1_ and Gα_s_ [20, 31]. These domains include the switch II region, switch III region, α3 helix, αG/α4 loop, α4 helix and the α4/β6 loop, and molecular modeling of Gα_i1_ revealed that they may provide a planar surface for protein–protein interaction (Fig. 1A). It is likely that Gα_i1_ employs one or more of these regions to interact with AC.

**Fig. 2:**
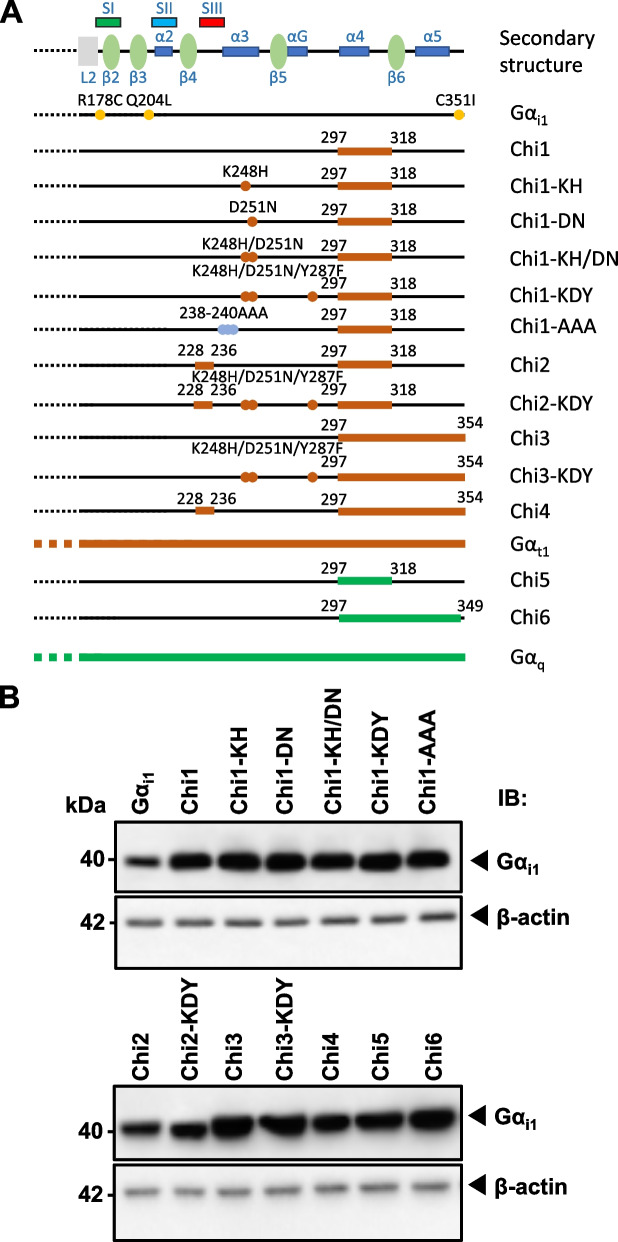
Construction and expression of Gα_i1_ chimeras. **A** Homologous replacements or point mutations on putative effector-interacting domains were made between Gα_i1_ (black) and Gα_t1_ (orange) (Chi1-4) or Gα_q_ (green) (Chi5 and Chi6). Sites of replacement/mutation are indicated by their residue numbers. The locations of GTPase-deficient mutations, namely R178C and Q204L, and PTX-insensitive mutation (C351I) are highlighted with yellow dots. **B** Expression of the chimeras was verified by Western blotting. HEK293 cells in a 24-well plate were transfected with 0.2 μg of various chimeric constructs and the cell lysates were subjected to immunoblotting using antibodies against Gα_i1_ and β-actin. Expressions of the chimeras were compared with that of Gα_i1_

Since Gα_t1_ and Gα_q_ share approximately 60% homology with Gα_i1_ but do not interact with AC, they have been proven as suitable partners for generating chimeras with Gα_i_ subunits [17, 27]. A series of Gα_i1_ chimeras were constructed (Fig. 2A) with one or more of their putative effector recognition domains substituted by homologous regions of Gα_t1_ (Chi1-4) or Gα_q_ (Chi5-6). We began by swapping the entire α4 helix to the α4/β6 loop of Gα_i1_ (residues 297–318) with the homologous region of Gα_t1_ to form Chi1 (referred to as Chi3 in [27]) (Fig. 2A). This domain was previously demonstrated to be important for AC inhibition by Gα_i2_ [17, 18] and Gα_z_ [19]. Chi2 was created by an additional swapping in the switch III region (referred to as Chi7 in [27]). This chimera was found to interact with phosphodiesterase γ (PDEγ) as efficiently as Gα_t1_ [27], and therefore may have switched its effector preference from AC into PDEγ. Chi3 was constructed with the Gα_t1_ sequence in Chi1 extended up to the C-terminus (Fig. 2A) because an equivalent chimera (named as zt295) using Gα_z_QL as the backbone resulted in a loss of the constitutive AC inhibition [19]; the AC-inhibiting surface of Gα_i1_ might be similarly affected in Chi3. Chi4 (also referred to as Chi4 in [27]), was designed such that both the switch III region and the C-terminal region starting from the α4 helix of Gα_i1_ were swapped with that of Gα_t1_ (Fig. 2A). Similar to Chi2, this chimera was previously shown to interact with PDEγ, which suggests that the effector specificity of the Chi4 is geared towards PDEγ [27]. Chi5 and Chi6 were equivalent to Chi1 and Chi3, except Gα_q_ sequence was used to replace the targeted segments of Gα_i1_ (Fig. 2A). As Gα_q_ has a lower overall homology to Gα_i1_ than Gα_t1_ [29], it is expected that such replacement would be more effective than Gα_t1_ in abolishing the activity of the GTPase-deficient mutations. In addition, Chi6 has retained the last 5 residues of the Gα_i1_. Retainment of the last 5 residues of Gα_i_ would allow subsequent examination of the chimera for activation by G_i_-coupled receptor [32].

We have additionally incorporated several point mutations that have previously been found to be important for effector interactions in selected chimeras (Figs. 1B and 2A). Two residues on the α3 helix of Gα_t1_ (H244 and N247; equivalent to K248 and D251 in Gα_i1_) are critical albeit not sufficient for conferring its activity [28], but full activity can be attained in association with another residue (F283) on the αG/α4 loop [21]. Since this latter residue is also critical for the stimulatory activity of Gα_s_ [21], it may represent an important determinant for interaction between Gα_t1_/PDEγ and Gα_s_/AC. Unlike Gα_t1_ and Gα_s_, Gα_i1_ possesses the more polar Y287 at the corresponding location (Fig. 1B). Hence, combinatorial replacement of K248, D251, and Y287 by cognate residues of Gα_t1_ (Fig. 2A) may impair the AC-inhibiting ability of the Gα_i1/t1_ chimeras. Another study on the effector-interacting domain of Gα_q_ revealed the importance of three consecutive residues in the switch III region (DNE motif, homologous to EEM in residues 238–240 of Gα_i1_) [33]. Owing to a conserved structure across all Gα subunits, it is possible that AC interaction will be eliminated when these three residues on Chi1 are all substituted by alanine (resulting in Chi1-AAA; Fig. 2A). All chimeras were expressed at levels comparable to parental Gα_i1_ in transiently transfected HEK293 cells (Fig. 2B).

### Constitutive activity of Gα_i1_RC is abolished by replacement of putative AC-interacting domains of Gα_i1_

**Fig. 3:**
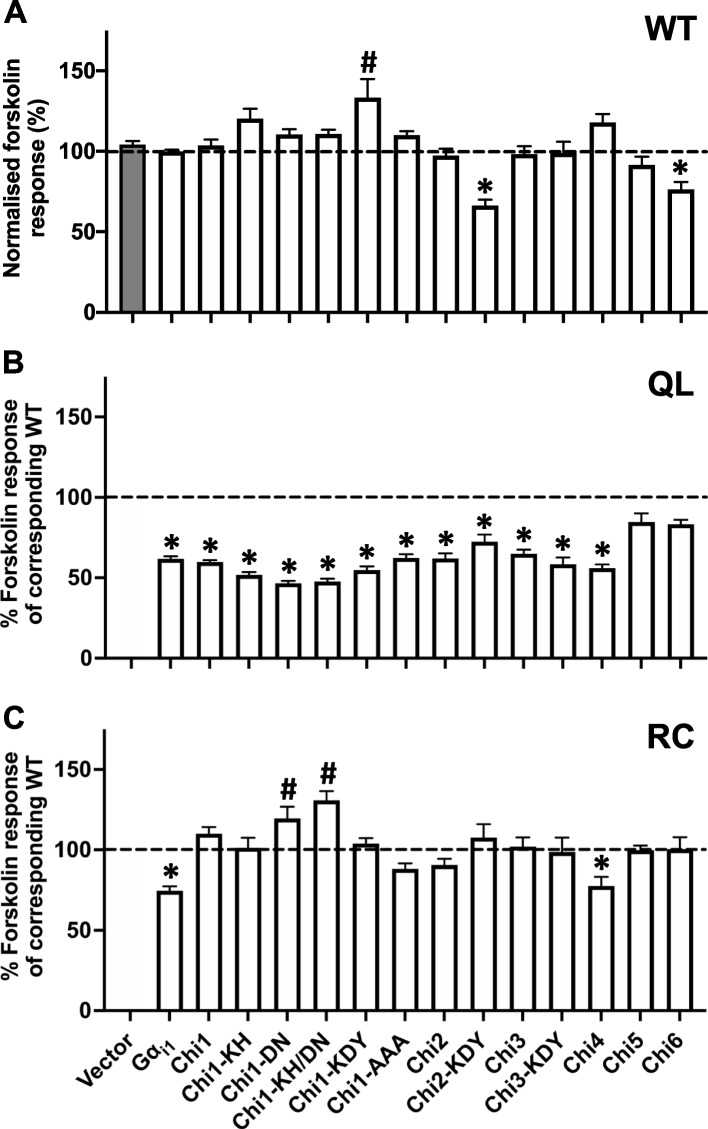
Effect of the QL/RC-bearing Gα_i1_ chimeras on forskolin-induced cAMP accumulation. HEK293 cells were transfected with 0.4 μg/mL of various chimeric constructs, labeled with [^3^H]adenine, and then assayed for [^3^H]cAMP accumulation in the presence of 50 μM forskolin. **A** Responses of the chimeras in WT version, as well as cells transfected with empty vector control (gray bar), towards forskolin were normalized against that of Gα_i1_. *, significantly lower than Gα_i1_; #, significantly higher than Gα_i1_. **B**, **C** The relative activities of the QL (**B**) or RC (**C**) chimeras are expressed as a percentage of cAMP accumulation of their corresponding WT. *, significantly lower than the corresponding WT, #, significantly higher than the corresponding WT. Data shown are mean ± SEM (*n* = 3). Bonferroni *t* test, *p* < 0.05

To test the effects of substitutions/mutations on the function of Gα_i1_, chimeras with or without either a QL or RC mutation were transfected into HEK293 cells, followed by the measurement of forskolin-induced [^3^H]cAMP accumulation. Three chimeric constructs, namely Chi1-KDY, Chi2-KDY and Chi6, showed constitutive stimulation/inhibition of AC activity without the incorporation of QL or RC mutations (Fig. 3A). Both Gα_i1_QL and Gα_i1_RC mutants suppressed cAMP elevation by forskolin to approximately 60% of the level observed with Gα_i1_ (Fig. 3B and C), consistent with previous findings indicating their constitutive activity [34–36]. Interestingly, as compared to the wild-type chimeras, none of the substitutions with Gα_t1_ affected the ability of the QL chimeras to inhibit AC (Fig. 3B). Yet, most of the RC chimeras (except Chi4RC) have lost the ability to inhibit cAMP production (Fig. 3C). It is noteworthy that purified Chi2 and Chi4 (referred to as Chi7 and Chi4 respectively in [27]) bind PDEγ as efficiently as an activated Gα_t1_ [27], but Chi2QL and Chi4QL/RC remained able to inhibit AC when overexpressed in cells (Fig. 3B). Our findings clearly showed functional differences between Gα_i1_QL and Gα_i1_RC (albeit both are constitutively active) *in cellulo*. Apparently, the activity of Gα_i1_RC can be more easily compromised by chimeric manipulations. A summary of their inhibitory activities towards AC is shown in Table 1.

**Table 1:** Activities of QL-/RC-bearing chimeras towards forskolin response

	cAMP level (% of Gα_i1_)		
Gα	WT	QL	RC
Gα_i1_	100.0 ± 1.5	57.7 ± 1.5*	70.9 ± 4.9*
Chi1	103.6 ± 3.7	69.0 ± 2.4*	117.9 ± 4.5
Chi1-KH	120.2 ± 6.2	62.2 ± 2.8*	124.8 ± 3.6
Chi1-DN	110.4 ± 3.2	52.5 ± 2.5*	135.0 ± 8.3^#^
Chi1-KH/DN	110.9 ± 2.4	55.7 ± 2.0*	149.3 ± 7.9^#^
Chi1-KDY	133.3 ± 11.6^†^	55.7 ± 2.9*	136.9 ± 8.8^#^
Chi1-AAA	110.0 ± 2.5	68.5 ± 2.3*	97.1 ± 3.6
Chi2	97.3 ± 4.2	59.6 ± 1.6*	87.6 ± 3.3
Chi2-KDY	66.2 ± 3.9^^^	47.3 ± 3.8*	67.0 ± 1.8
Chi3	98.2 ± 4.9	63.7 ± 3.4*	98.5 ± 2.4
Chi3-KDY	98.9 ± 7.1	57.7 ± 4.1*	97.4 ± 8.6
Chi4	117.9 ± 5.3	70.3 ± 4.6*	90.9 ± 6.4*
Chi5	91.5 ± 5.0	78.0 ± 6.2	90.8 ± 2.9
Chi6	76.2 ± 4.7^^^	66.4 ± 3.7	82.2 ± 3.2

HEK293 cells overexpressing the chimeric constructs were subjected to cAMP accumulation assay. Percentage of Gα_i1_ was calculated by the fraction of forskolin-stimulated cAMP level in Gα_i1_-overexpressing cells. Data are shown as mean ± SEM (*n* = 3). The cAMP levels of QL/RC-bearing chimeras were compared to the chimeras of the wild-type (WT) version. Datum with an asterisk (*) indicates the cAMP level is significantly lower than the WT control, while datum with a hashtag (#) indicates the cAMP level is significantly higher than the WT control. The cAMP levels of chimeras of the WT version were also compared with that of Gα_i1_. Datum with (†) indicates an elevated basal cAMP level, while datum with (^) indicates a lower basal cAMP level. Bonferroni *t* test, *p* < 0.05

Chi6 appeared to inhibit AC constitutively (Fig. 3A). As the C-terminus of Gα_q_ is important for effector interaction [33], we sought to test if its effector specificity has been switched to PLCβ which may then indirectly inhibit AC activity [37]. Chi6QL did not stimulate the production of inositol phosphates (IP) whereas constitutively active Gα_q_QL significantly stimulated the PLCβ activity under the same experimental condition (Fig. S1A), suggesting that Chi6 cannot activate PLCβ.

### Activity-compromised Gα_i1_ chimeras can suppress cAMP level upon receptor activation

**Fig. 4:**
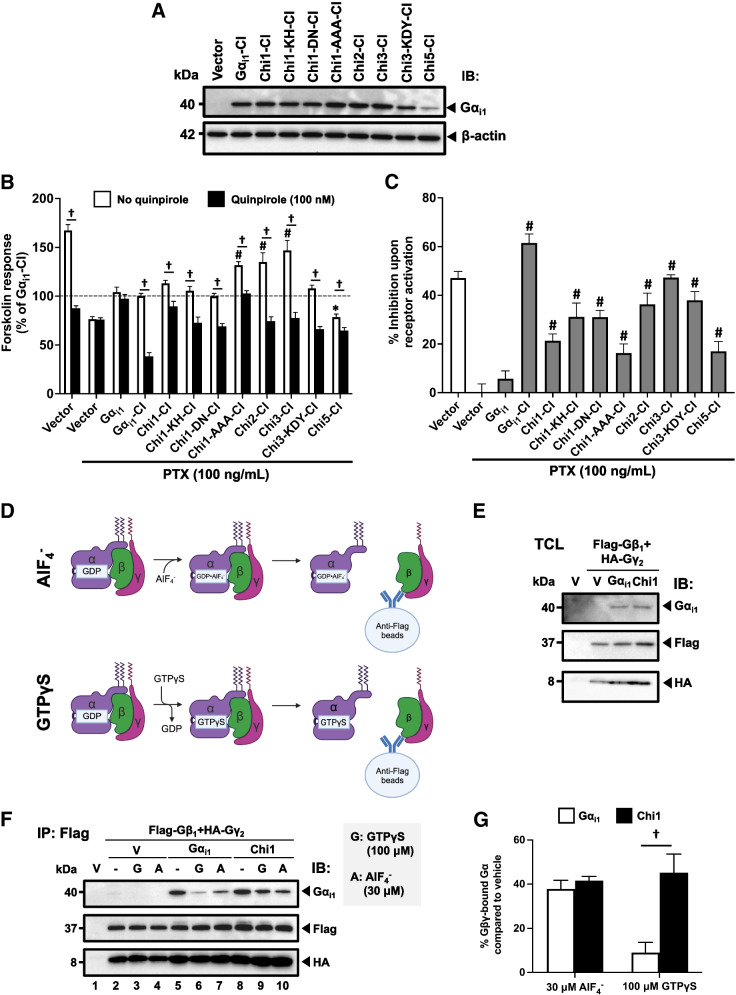
Activity of PTX-insensitive Gα_i1_ chimeras upon receptor activation. HEK293 cells were co-transfected with D_2_R and various Gα_i1_ constructs (0.2 μg/mL each), treated with PTX (100 ng/mL, 16 h), and then assayed for forskolin-induced [^3^H]cAMP accumulation in the absence or presence of 100 nM quinpirole. **A** Expression of the PTX-insensitive Gα_i1_ chimeric mutants was confirmed by immunoblotting with 20 μg of total protein. **B** Forskolin-stimulated cAMP levels are expressed as a percentage of the response normalized against Gα_i1_-CI. **C** Quinpirole-induced activity is expressed as a percentage of inhibition of the forskolin response. Data shown are mean ± SEM (*n* = 3). Bonferroni *t* test, *p* < 0.05; *, significantly lower than the control; #, significantly higher than the control; †, significant inhibition upon receptor activation. **D** Rationale of the subunit dissociation assay. Activated Gα_i1_ dissociates with Gβγ, resulting in a drop in Gα_i1_ intensity in immunodetection after co-immunoprecipitation with the Flag-tagged Gβ. Gα_i1_ activation by GTPγS, but not AlF_4_^−^, requires guanine nucleotide exchange. **E**–**G** HEK293 cells were transiently co-transfected with 0.2 μg/mL each of Flag-tagged Gβ_1_, HA-tagged Gγ_2_, and either vector (V), Gα_i1_ or Chi1. **E** Expressions of the G proteins were confirmed by immunoblotting with 20 μg of the total proteins. **F** 500 μg of the total proteins of the lysate were incubated with or without AlF_4_^−^ (30 μM AlCl_3_ plus 10 mM NaF) or 100 μM of GTPγS at 37 °C for 15 min prior to immunoprecipitation by anti-Flag affinity gel. **G** Quantification of the co-immunoprecipitation results. Results are expressed as a percentage of Gα_i1_ or Chi1 pull-down by Flag-Gβ_1_. Graph is shown as mean ± SEM (*n* = 3). Student *t* test, *p* < 0.05; †, significantly different

In the preceding experiments, many RC-bearing chimeras lost their ability to inhibit AC while most of the chimeric QL mutants remained able to suppress the forskolin response (Fig. 3 and Table 1). The contrasting results obtained with the QL and RC mutants of the chimeras implied that there may be discernable differences in the active conformations promoted by these two mutations. We thus examined which of the two mutants have a closer resemblance to Gα_i1_ activated by a receptor, with the latter being more biologically relevant. We determined the chimeras’ ability to mediate receptor-induced inhibition of cAMP accumulation. To enable detection of receptor-mediated responses without interference from endogenous G_i_ proteins, a C351I (CI) mutation was introduced into the chimeras to provide resistance to PTX [38]. Eight chimeras that exhibited differential abilities to abolish the constitutive activities of the QL or RC mutation were selected and their corresponding CI mutants constructed; with the exception of Chi5-CI, these chimeras were expressed at levels comparable to that of the Gα_i1_-CI mutant (Fig. 4A). HEK293 cells co-expressing the G_i_-coupled dopamine D_2_ receptor (D_2_R) and a chimera with the CI mutation were pretreated with PTX before assaying for forskolin-induced cAMP accumulation in the absence or presence of 100 nM of quinpirole (agonist for D_2_R). PTX treatment effectively inhibited the ability of Gα_i1_ to be activated by D_2_R (Fig. 4B), hence any detected suppression of cAMP level would be primarily due to the activity of the PTX-resistant chimeras. The positive control, Gα_i1_-CI, produced ~ 60% inhibition of forskolin-induced cAMP response upon activation by the receptor (Fig. 4B and C). Surprisingly, all CI chimeras significantly inhibited AC upon D_2_R activation (Fig. 4B), albeit weaker than that of Gα_i1_-CI (Fig. 4C). The extent of inhibition varied among the chimeras, with a maximum of 50% inhibition observed with Chi3-CI, while Chi1-AAA-CI and Chi5-CI only produced ~ 20% inhibition (Fig. 4C). Chi1-AAA-CI, Chi2-CI and Chi3-CI had an elevated cAMP level upon treatment with forskolin, ranging from a 30% to 50% increase (Fig. 4B).

Because GDP/GTP exchange on the Gα subunit triggered by an activated receptor is initiated from the C-terminal end of the Gα subunit to the switch regions [39], alterations in the C-terminal half of Gα_i1_, as in the chimeras, may affect the rate of guanine nucleotide exchange, thereby attenuating its ability to inhibit AC. To test if Chi1, a prototypical chimera, can adopt the active conformation as efficiently as Gα_i1_, we examined GTP-induced release of Gβγ in HEK293 cells co-expressing Flag-tagged Gβ_1_ and HA-tagged Gγ_2_ with Chi1 or Gα_i1_ (Fig. 4D). Lysates were treated with either aluminum fluoride (AlF_4_^−^) or GTPγS to activate the Gα subunits. AlF_4_^−^ acts as a mimetic of the γ-phosphate of GTP in GDP•AlF_4_^−^-bound Gα subunits, and it can thus activate Gα subunits without requiring guanine nucleotide exchange (Fig. 4D) [40]. GTPγS is a non-hydrolyzable analog of GTP which locks the Gα subunit into an active conformation upon guanine nucleotide exchange (Fig. 4D) [41]. Activated Gα_i1_ should dissociate from the Gβγ dimer and thus would not co-immunoprecipitate with the Flag-tagged Gβ_1_ subunit (Fig. 4D). Expression of the different G protein subunits in the transfectants was confirmed by Western blots (Fig. 4E). The HA-tagged Gγ_2_ was efficiently co-immunoprecipitated with Flag-Gβ_1_, in line with Gβγ being a constitutive dimer in cells. As shown in Fig. 4F (lanes 5 and 8), both Gα_i1_ and Chi1 were pulled down by anti-Flag affinity beads along with the Flag-tagged Gβ_1_ subunit. Upon treatment with GTPγS, almost all Gα_i1_ dissociated from the Gβγ dimer (Fig. 4F, lane 6), but a substantial portion of Chi1 remained associated with the Gβγ dimer (Fig. 4F, lane 9); the extent of co-immunoprecipitation was quantified in Fig. 4G. In contrast, AlF_4_^−^ treatment resulted in the dissociation of ~ 60% of the Gβγ-bound Gα_i1_ and Chi1, suggesting that Chi1 can adopt an active conformation similar to Gα_i1_ (Fig. 4F and G). Since the effect of GTPγS requires the release of bound GDP from the Gα subunit while the action of AlF_4_^−^ is independent of such an event, these results indicate that the rate of guanine nucleotide exchange of Chi1 may be impaired, leading to apparent reductions in the AC inhibitory activity of the chimeras. This also implies that the loss of activity of RC chimeras is not due to their inability to interact with the downstream effector. Instead, the GTPase deficiency brought about by RC mutation is compromised.

Although Chi5 showed no inhibitory effect on cAMP level (Fig. 3A), Chi5-CI appeared to constitutively inhibit the forskolin-stimulated cAMP accumulation (Fig. 4B), and the forskolin response was further suppressed upon D_2_R-induced activation of Chi5-CI (Fig. 4C). Given that Chi5-CI contains the PLCβ-activating domain of Gα_q_ [33], we examined if this chimera could generate IP_3_/Ca^2+^ signals via G_q_. Quinpirole-induced IP formation was readily observed with Gα_qz5_ (positive control) [32] but not with Chi5-CI (Fig. S2A). Gα_qz5_ also showed a typical dose–response curve on Ca^2+^ mobilization upon D_2_R stimulation, with the maximum signal observed at 100 nM quinpirole (Fig. S2B). Yet, Chi5-CI did not stimulate Ca^2+^ mobilization even at 10 μM quinpirole (Fig. S2B). Therefore, Chi5-CI did not stimulate the G_q_ signaling pathway.

### Gα_i1_RC-CI can respond to receptor activation

**Fig. 5:**
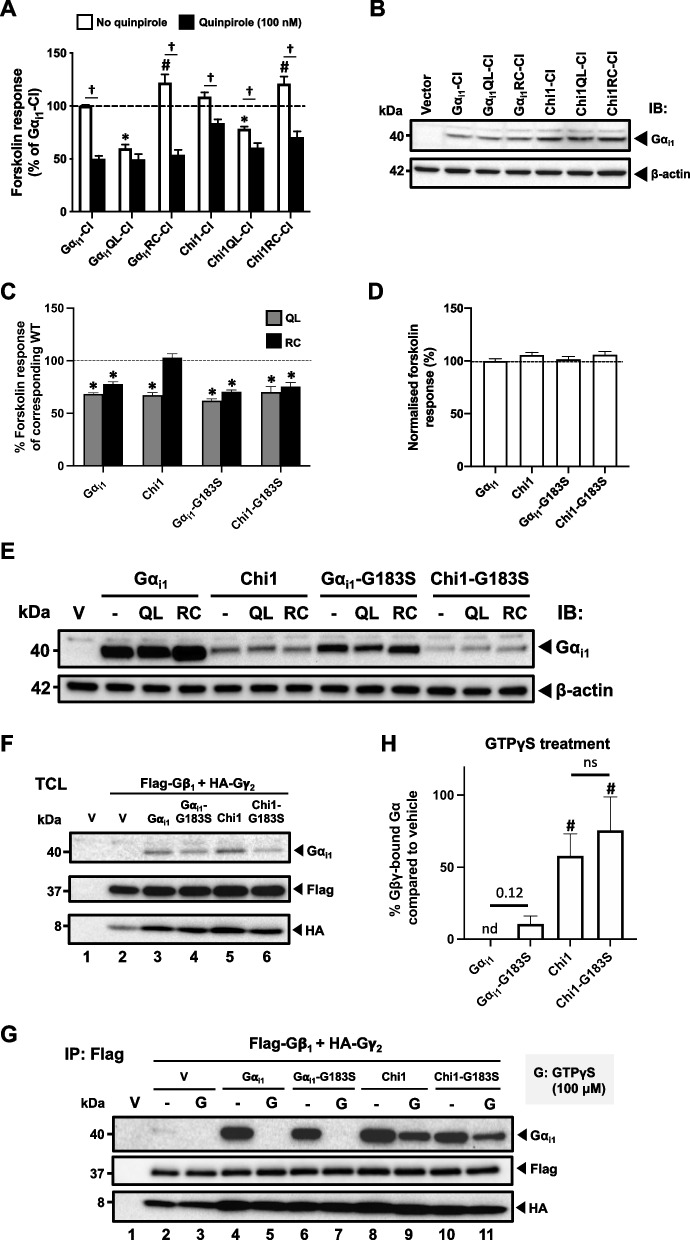
RC mutants can be activated by receptor and suppressed by RGS. **A** HEK293 cells were co-transfected with D_2_R and various Gα_i1_-CI or Chi1-CI mutants and assayed similarly to Fig. 4B. The forskolin-stimulated cAMP levels of the chimeras with a CI mutation are expressed as a percentage of the response obtained with Gα_i1_-CI. Data shown are mean ± SEM (*n* = 3). Bonferroni *t* test, *p* < 0.05; *, significantly lower than the control; #, significantly higher than the control; †, significant inhibition upon receptor activation. **B** Expression of the PTX-insensitive mutants was confirmed by immunoblotting with 20 μg of total protein. **C**-**E** HEK293 cells were transfected with QL-bearing Gα_i1_ constructs and assayed similarly to Fig. 3. **C** Relative activities of the constitutively active chimeras are expressed as a percentage of cAMP accumulation of their corresponding WT. *, significantly lower than the corresponding WT; #, significantly higher than the corresponding WT. Data shown are mean ± SEM (*n* = 3). Bonferroni *t* test, *p* < 0.05. **D** Responses of the chimeras in WT version towards forskolin were normalized against that of Gα_i1_. **E** Expression of Gα_i1_ constructs were confirmed by immunoblotting with 20 μg of total protein. **F**–**H** HEK293 cells were transiently co-transfected with Flag-Gβ_1_, HA-Gγ_2_, and with or without various Gα_i1_ constructs and assayed by subunit dissociation assay as in Fig. 4D. **F** Expressions of the G proteins were confirmed by immunoblotting with 20 μg of the total proteins. **G** 500 μg of the total proteins of the lysate were incubated with or without 100 μM of GTPγS at 37 °C for 15 min prior to immunoprecipitation by anti-Flag affinity gel. **H** Quantification of the co-immunoprecipitation results. Results are expressed as a percentage of the corresponding Gα_i1_ or Chi1 constructs pull-down by Flag-Gβ_1_. Graph shown as mean ± SEM (*n* = 3). Student *t* test, *p* < 0.05; n.d., not detectable; ns, non-significant; #, significantly higher than the control

The ability of D_2_R to activate CI-bearing chimeras and suppress the forskolin response (Fig. 4) indicated that these chimeras still contain the necessary domains for interacting with AC. This also explains the inhibitory actions as observed with the chimeric QL mutants (Fig. 3 and Table 1). The lack of constitutive activity of the corresponding RC mutants, however, suggested that the active conformation of these Gα_i1_ chimeras cannot be efficiently induced and/or maintained. Hence, we asked if Gα_i1_QL and Gα_i1_RC would respond differently to receptor activation. The CI mutation was introduced into Gα_i1_QL and Gα_i1_RC and the resultant mutants, named as Gα_i1_QL-CI and Gα_i1_RC-CI, were co-expressed with D_2_R in HEK293 cells and then subjected to PTX treatment prior to assaying for forskolin-stimulated cAMP accumulation. In the absence of quinpirole, Gα_i1_QL-CI significantly suppressed the cAMP level to 50% of that obtained with the control (Gα_i1_-CI; Fig. 5A). This constitutive activity of Gα_i1_QL-CI was similar to that of Gα_i1_-CI-mediated AC inhibition upon D_2_R activation by quinpirole, indicating attainment of maximal inhibitory activity. However, cells co-transfected with D_2_R and Gα_i1_RC-CI produced an unexpected 20% increase in the forskolin response (Fig. 5A). In the presence of quinpirole, Gα_i1_RC-CI significantly inhibited the forskolin response by over 55% (Fig. 5A), thus suggesting that Gα_i1_RC-CI can interact with the receptor. This observation is important because it eliminates several possibilities that might account for the loss of AC-inhibitory ability of Gα_i1_RC-CI when co-expressed with D_2_R. Firstly, as the PTX-insensitive mutants showed similar expression levels (Fig. 5B), the lack of AC inhibition by Gα_i1_RC-CI was not attributed to decreased expression of this mutant. Secondly, the ability of quinpirole-treated Gα_i1_RC-CI-expressing cells to suppress forskolin-induced cAMP elevation to a level similar to Gα_i1_-CI upon receptor activation (Fig. 5A) suggested that Gα_i1_RC-CI can adopt an active conformation, allowing its interaction with AC. As Cys-351 is distant from the nucleotide binding pocket of Gα_i1_RC [42], it is unlikely that the CI mutation would directly participate in GTP hydrolysis to inactivate Gα_i1_RC-CI.

Next, we examined if the loss of activity of RC chimeras is due to their failure to maintain, or alternatively, induce the active conformation of the Gα subunit. To test this, we introduced the CI mutation to Chi1QL and Chi1RC, the prototypical chimeric constructs. Chi1-CI showed approximately 25% suppression of cAMP level upon receptor activation (Fig. 5A). Chi1QL-CI was constitutively active without quinpirole treatment, with the forskolin response reduced to a level similar to an activated Chi1-CI (Fig. 5A). Receptor activation enhanced the inhibition on cAMP level by Chi1QL-CI, suggesting Chi1QL-CI is not fully active (Fig. 5A). Like Gα_i1_RC-CI (Fig. 5A), Chi1RC-CI did not inhibit the forskolin-induced cAMP accumulation and showed prominent AC inhibition only upon quinpirole treatment, indicating that the active conformation of Chi1RC-CI is inducible (Fig. 5A). Thus, the loss of AC inhibition by RC chimeras may be attributed to the lack of maintenance of their active conformation.

### Chi1RC is RGS-sensitive in cellulo

Since Gα_i1_RC-CI and Chi1RC-CI could be activated by D_2_R (Fig. 5A), it implies that they may adopt an inactive conformation in the absence of receptor activation despite harboring the RC mutation. Because an active GTP•Gα_i1_ has a low affinity for the receptor [43], it further suggests that a substantial portion of the Gα_i1_RC-CI is GDP-bound. Given that the RC mutation impairs the intrinsic GTPase activity [44], the GDP-bound state (as opposed to a GTP-locked state) can be obtained by two means: the prevention of GDP/GTP exchange by guanine nucleotide dissociation inhibitors, and the extrinsic promotion of GTP hydrolysis by GTPase-activating proteins (GAPs). An early reconstitution study showed that RGS4 could promote the GTP hydrolysis of Gα_i1_RC, but not for Gα_i1_QL [24], although *in cellulo* evidence remains lacking. Thus, the lack of constitutive activity of RC chimeras may be attributed to their interaction with RGS proteins which aids in maintaining the GDP-bound state of the Gα subunits. To test this hypothesis, we incorporated an RGS-insensitive G183S mutation [45] into QL/RC-bearing Gα_i1_ and Chi1, and then examined their AC inhibitory activities. As shown in Fig. 5C, both QL and RC versions of Gα_i1_-G183S constitutively suppressed cAMP accumulation to an extent similar to Gα_i1_QL and Gα_i1_RC, respectively. It is also worth noting that G183S mutation alone did not produce any effect on AC inhibition (Fig. 5D). These observations suggested that RGS proteins did not hinder the interactions between AC and the two constitutively active mutants. Both Chi1QL and Chi1QL-G183S produced significant AC inhibition (Fig. 5C). Strikingly, G183S mutation enabled Chi1RC to suppress cAMP production (Fig. 5C). Although mutants bearing the G183S mutation showed a lower expression (Fig. 5E), the level was nevertheless sufficient to generate a significant cAMP suppression (Fig. 5C). Collectively, the lack of AC inhibition by Chi1RC, and possibly other RC chimeras, might be attributed to ‘hyper’-interactions of the Gα subunits with RGS proteins. This also provides the first *in cellulo* evidence that RC mutation is RGS-sensitive.

As Chi1RC-G183S inhibits AC to the same extent as Gα_i1_RC (Fig. 5C), one would expect activated Chi1-G183S to dissociate from the Gβγ dimer like a Gα_i1_. However, it is also possible that RGS proteins may displace the Gβγ dimer from an active Gα_i1_. Co-crystal structures of RGS-Gα_i1_ reveal that RGS proteins bind orthogonally to the switch regions of Gα_i1_ [25, 46]. In fact, RGS4 inhibited Gα_q_-mediated activation of PLCβ1 by direct blockade of the binding interface [47]. As Gβγ dimer covers the switch regions of Gα_i1_ in its inactive, heterotrimeric state [48], RGS proteins may compete with Gβγ dimer for binding Gα_i1_. In this case, an activated Chi1-G183S will have a higher association with Gβγ dimer than Chi1, because the G183S mutation prevents Chi1 from binding to RGS proteins [45]. Therefore, we treated lysates of cells expressing Chi1 or Chi1-G183S and Gβ_1_γ_2_ with GTPγS and tested for their dissociations with Gβγ dimer. All subunits were well expressed (Fig. 5F). Interestingly, G183S did not affect the extent of dissociation of Gβ_1_γ_2_ from either Gα_i1_ or Chi1 upon GTPγS treatment (Fig. 5G and H , lanes 5 vs 7 and lanes 9 vs 11). This implies that RGS proteins may form a transient quaternary complex with Gα_i1_ and Gβγ dimer to elicit its GAP activity.

### Gα_i1_ retains its AC inhibitory capability with known switch II mutations

**Fig. 6:**
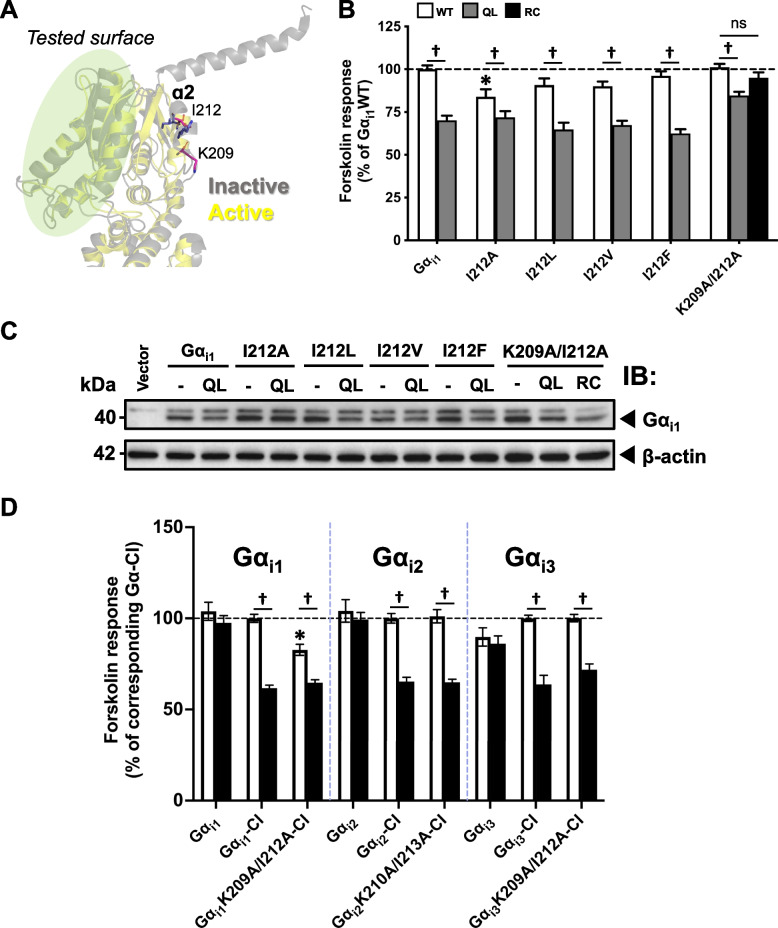
Effect of K209 and I212 mutations on the AC inhibition QL/RC. **A** Expanded view of the 3-dimensional structure of Gα_i1_ highlighting the tested surface (in light green) and the location of K209 and I212 (in sticks) at the α2 helix. **B** HEK293 cells were transfected with various QL/RC-bearing Gα_i1_ constructs and assayed similarly to Fig. 3. The relative activities of the constitutively active chimeras are expressed as a percentage of cAMP accumulation of Gα_i1_. Data shown are mean ± SEM (*n* = 3). Bonferroni *t* test, *p* < 0.05; ns, non-significant; †, significant inhibition. **C** Expression of Gα_i1_ constructs were confirmed by immunoblotting with 20 μg of total protein. **D** HEK293 cells were co-transfected with D_2_R and various Gα_i1-3_ mutants and assayed as in Fig. 4B. The forskolin-stimulated cAMP levels of the chimeras with a CI mutation are expressed as a percentage of the response normalized with the corresponding Gα-CI. Data shown are mean ± SEM (*n* = 3). Bonferroni *t* test, *p* < 0.05; *, significantly lower than the control; †, significant inhibition upon receptor activation

The failure of the chimeras to abolish receptor-induced cAMP suppression (Fig. 4B and C) suggests that there exists an alternative and largely unstudied surface of Gα_i1_ which participates in the inhibition of AC. In fact, the α2 helix, which is distally located to the other documented domains (Fig. 6A), was found to be critical for AC interactions of Gα_i2_ and Gα_s_ [18]. The α2 helix is part of the switch II region, which has extensive conformational changes upon activation of the Gα subunit (Fig. 6A). In particular, double alanine mutations on K210/I213 of Gα_i2_ and the homologous R232/I235 of Gα_s_ (corresponding to K209/I212 of Gα_i1_) can eliminate the constitutive activity of their respective RC mutants [18]. Considering the results shown in Figs. 3 and 4, we examined the effect of K209/I212 mutations on Gα_i1_. Distinct conformational changes in the side chain orientation of I212, specifically a shifting from a protein-core pointing to an outward pointing configuration during the transition from inactive to active state [42, 48], have been observed, suggesting its potential role in AC interaction. However, the I212A mutant exhibited AC inhibitory activity and did not abrogate the constitutive activity of QL (Fig. 6B). We also substituted I212 with other residues, including leucine (L) and valine (V) to maintain comparable molecular size of the side chain, so that the structural perturbation of the mutations can ideally be minimized. The activity of I212F-QL was also examined, with Phe being the analogous residue in non-AC-interacting Gα_12/13_. Interestingly, all I212 substitutions tested failed to suppress the constitutive activity of QL mutation (Fig. 6B) in spite of their comparable expression levels with Gα_i1_QL (Fig. 6C). Double K209A/I212A mutation only abolished the activity of RC, but not the QL activity (Fig. 6B). Moreover, cAMP suppressions were still observed from Gα_i1-i3_-CI bearing the double mutations upon activation by D_2_R (Fig. 6D). This is consistent with our observations that chimeras which failed to abolish the constitutive activity of QL remained activatable by receptors (Fig. 4). This also supports our notion that RC may not be the best representative of an activated Gα_i_, because a loss of constitutive activity of RC was similarly observed with Gα_i2_ bearing the cognate mutations (Gα_i2_K210A/I213A-RC; [18]).

### Gα_i1_QL remains constitutively active with mutations that target potential AC-interacting residues

We have also investigated other potentially novel sites for interaction with AC (Fig. S3). The proximity of positively charged residues in Gα_i1_ near switch II (K35, H188, and K197) suggests their potential to form charge-charge interactions with AC5/AC6. Additionally, the E489 residue located in the C1 domain of the AC5 protein (Uniprot: O95622-1), known for its role in Gα_i_-mediated AC inhibition [49], holds promise for forming specific charge-charge interactions with these residues within Gα_i1_. Similarly, E216 and K257 in Gα_i1_ may play a role in the interaction with AC in view of known interactions between the homologous N239 and R280 in Gα_s_ and the AC9 protein [50]. We generated a double alanine mutant (E216A/K257A) and an E216K/K257E mutant to explore the significance of the charge interactions between these two residues of Gα_i1_ and AC. Previous research revealed that mutation in the αG-α4 loop significantly impacts the stimulatory activity of the Gα_s_, despite its spatial distance from the switch II [21]. This suggests that the corresponding loop in the Gα_i1_, akin to Gα_s_, may interact with AC. To explore this further, we substituted residues PLT (282–284) in the αG-α4 loop of Gα_i1_ with the HLS residues from Gα_t1_, creating the mutant termed PLT. These mutants were evaluated for the possible loss of AC inhibition function (Fig. S3). However, all QL-bearing mutants with additional mutations at the described residues remained capable of suppressing forskolin-induced cAMP accumulation (Fig. S3), reflecting that these residues/regions are not critical to AC interaction by Gα_i1_.

### Additional mutations on the α3/β5 loop synergistically abolished receptor-induced AC inhibition of Gα_i1-i3_ with the α4/β6 loop

Although point mutations on the α2 helix of Gα_i1_ failed to entirely eliminate the ability of Gα_i1_ to inhibit AC (Fig. 6), it remains possible that adjacent regions may participate in effector recognition. The α2 helix is almost completely covered by Gβ subunit in the GDP-bound inactive state [48], and it undergoes extensive conformational changes upon GTP binding [42]. The subsequent Gβγ release exposes surfaces encompassing the α2 helix. Thus, it is likely that the surrounding residues could be important for effector recognition.

The challenge of our investigation lies in the lack of a crystal structure of the Gα_i1_-AC complex. To circumvent this limitation, we employed High Ambiguity Driven protein − protein DOCKing (HADDOCK) to simulate the interactions between a well-resolved structure of an active Gα_i1_ and the AlphaFold-simulated structures of human AC5 and AC6, the two AC subtypes known to interact with Gα_i1_ [51, 52]. K208, K209, and I212 of Gα_i1_ were previously designated as "active" residues in interacting with ACs [18]. For AC5, we selected E489, M492, T493, L550, and V554 of the C1 domain (and E399, M402, T403, L460, and V464 for AC6) as "active" residues [49]. The simulations resulted in 174 predicted Gα_i1_-AC5 structures into 5 clusters based on similarity between individual models, representing 87% of the water-refined models (Table 2). Similarly, for the Gα_i1_-AC6 complex, HADDOCK predicted 139 structures in 9 clusters, constituting 69% of the generated models (Table 2). Given the higher reliability attributed to the top cluster with the lowest Z-score, we focused on investigating the structures from the leading cluster in each simulation.

**Table 2:** Parametric data on HADDOCK 2.4 predictions of Gα_i1_ and AC5/AC6 interactions

	HADDOCK score	Cluster size	RMSD	Van der Waals energy	Electrostatic energy	Desolvation energy	Restraints violation energy	Buried surface area	Z-score
**Gα**_**i1**_**-AC5**									
Cluster 1	-94.8 ± 2.5	84	19.4 ± 0.1	35.2 ± 5.6	-225.0 ± 29.5	-14.8 ± 2.5	2.0 ± 1.7	1367.6 ± 113.0	-0.9
Cluster 2	-93.7 ± 6.6	42	17.2 ± 0.2	-27.4 ± 4.1	-284.8 ± 17.7	-10.1 ± 3.8	7.1 ± 2.2	1433.4 ± 69.9	-0.8
Cluster 4	-88.0 ± 1.4	17	18.6 ± 0.2	-40.5 ± 2.1	-153.8 ± 24.0	-17.0 ± 4.2	3.0 ± 1.1	1287.7 ± 25.6	-0.2
Cluster 3	-84.7 ± 0.7	23	12.5 ± 0.2	-41.0 ± 4.6	-183.6 ± 41.4	-8.1 ± 3.9	11.7 ± 6.6	1537.2 ± 50.6	0.1
Cluster 5	-67.5 ± 7.8	8	1.2 ± 0.2	-23.6 ± 2.6	-217.2 ± 35.0	-2.5 ± 3.2	19.9 ± 18.9	1294.9 ± 59.1	1.9
**Gα**_**i1**_**-AC6**									
Cluster 1	-100.5 ± 9.8	70	1.6 ± 1.0	-42.0 ± 6.7	-234.4 ± 41.7	-13.5 ± 2.0	19.1 ± 16.7	1476.7 ± 91.4	-1.9
Cluster 2	-85.6 ± 4.6	21	17.8 ± 0.2	-45.5 ± 2.1	-208.8 ± 17.7	-0.6 ± 1.3	22.5 ± 19.1	1709.5 ± 60.7	-1.0
Cluster 3	-79.1 ± 4.1	12	9.3 ± 0.3	-37.5 ± 3.9	-242.3 ± 41.4	6.5 ± 2.1	3.6 ± 1.7	1333.6 ± 39.9	-0.7
Cluster 4	-68.6 ± 4.0	11	6.4 ± 1.1	-33.0 ± 4.4	-163.2 ± 33.9	-5.2 ± 3.5	22.5 ± 32.9	1184.0 ± 108.8	-0.1
Cluster 6	-64.3 ± 6.7	6	10.4 ± 0.3	-19.8 ± 2.1	-267.8 ± 35.5	6.7 ± 2.6	23.5 ± 10.5	1250.8 ± 119.6	0.1
Cluster 9	-57.6 ± 12.1	4	3.1 ± 0.2	-23.9 ± 4.8	-162.0 ± 34.5	-1.4 ± 1.7	2.0 ± 0.8	1212.2 ± 228.0	0.5
Cluster 7	-55.6 ± 6.1	5	5.6 ± 1.7	-28.1 ± 3.8	-166.0 ± 31.9	3.4 ± 3.0	22.8 ± 13.3	1166.9 ± 131.2	0.6
Cluster 5	-51.4 ± 9.1	6	14.8 ± 0.3	-25.8 ± 5.1	-106.6 ± 39.8	-5.3 ± 4.5	9.7 ± 12.7	1171.7 ± 107.0	0.8
Cluster 8	-37.3 ± 7.1	4	12.4 ± 0.1	-14.1 ± 5.0	-77.7 ± 22.1	-8.6 ± 3.5	9.0 ± 1.6	789.9 ± 123.1	1.6

**Fig. 7:**
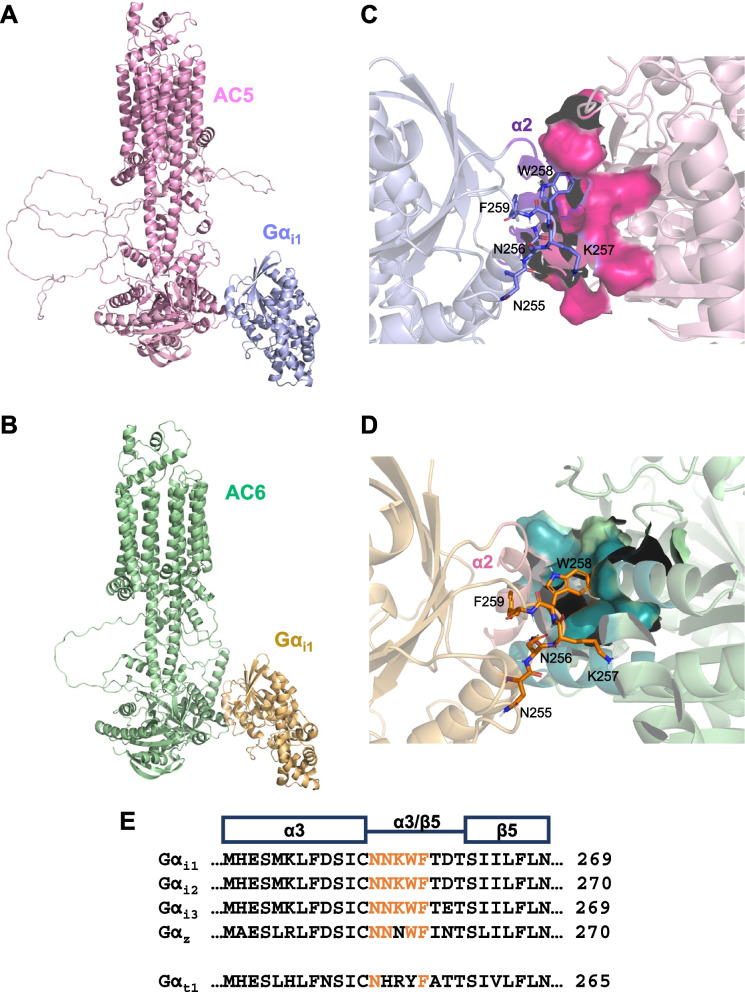
HADDOCK predictions on Gα_i1_-AC interactions. Three-dimensional structures on the best-scored predicted models showing **A** Gα_i1_-AC5 (in light blue and pink, respectively) and **B** Gα_i1_-AC6 (in wheat and green, respectively) interactions. Expanded views showing the interfaces of binding between Gα_i1_ and AC5 or AC6 were shown in (**C**) and (**D**) respectively. Locations of the key residues on the α3/β5 loop predicted to be important for such interactions are shown in sticks. **E** Sequence alignment of the region spanning the α3 helix to the β5 sheet of AC-inhibiting Gα, and Gα_t1_. Conserved residues are indicated in orange

The only crystal structure available for AC in complex with a Gα subunit is that of Gα_s_-AC9 [50]. Despite AC9 being unresponsive to inhibition by Gα_i_ [53], we utilized it as a benchmark to assess the reliability of structural predictions by HADDOCK. The predicted co-complexes of Gα_i1_-AC5 and Gα_i1_-AC6 generally exhibited a binding pattern reminiscent of the Gα_s_-AC9 structure (Fig. 7A and B). We observed a key interaction interface where the α2 helix of Gα_i1_ inserts into the groove formed by the α2 and α3 helices of the C1 domains of AC5 and AC6 (Fig. 7C and D), akin to the α2 helix of Gα_s_ interacting with the C2 domains of AC9. Similar binding modes were also evident in other top clusters, specifically clusters 2 and 4 for Gα_i1_-AC5, and clusters 4 and 9 for Gα_i1_-AC6 (data not shown). Subsequently, we analyzed the molecular interactions within the binding interfaces using PRODIGY and PDBsum. We identified 45 interactions between Gα_i1_ and AC5 and 58 interactions with AC6, which are comparable to Gα_s_-AC9 where 64 interactions were observed (Table 3). In the Gα_i1_-AC5 complex, interacting residues were clustered in several domains, including the α2 helix (R208-H213), the α2/β4 loop (F215-E216), the α3 helix (S252), and the α3/β5 loop (N255-W258) (Fig. S4). It was similarly observed for Gα_i1_-AC6, where the α2 helix (R205, R208-H213), the α2/β4 loop (F215), the α3 helix (L249, S252-I253), the α3/β5 loop (N256-W258), and the α4/β6 loop (D315-T316) were identified as sites of interaction (Fig. S4). These predictions are aligned with another molecular dynamic simulation study [54]. Notably, the α3/β5 loop is the only predicted region that was not previously studied. The predicted models indicated that the α3/β5 loop is coplanar to the α2 helix of Gα_i1_, and interacts with both AC5 and AC6 by forming multiple polar and apolar attractions with the key residues of the C1 domain (Figs. 7C, D and S4). Moreover, the α3/β5 loop is highly conserved in AC-inhibiting Gα_i1-3/z_ (Fig. 7E).

**Table 3:** PRODIGY predictions of intermolecular forces between Gα and AC isoforms

Complex	ΔG (kcal mol^−1^)	Kd(μM) at 37℃	Charged-charged interactions	Charged-polar interactions	Charged-apolar interactions	Polar-polar interactions	Polar-apolar interactions	Apolar-apolar interactions	Total no. of Interactions
Gα_i1_-AC5	-6.9	14.0	6	11	9	1	7	11	45
Gα_i1_-AC6	-8.6	0.93	9	11	20	1	8	9	58
Gα_s_-AC9	-9.3	0.29	6	7	17	2	14	18	64

**Fig. 8:**
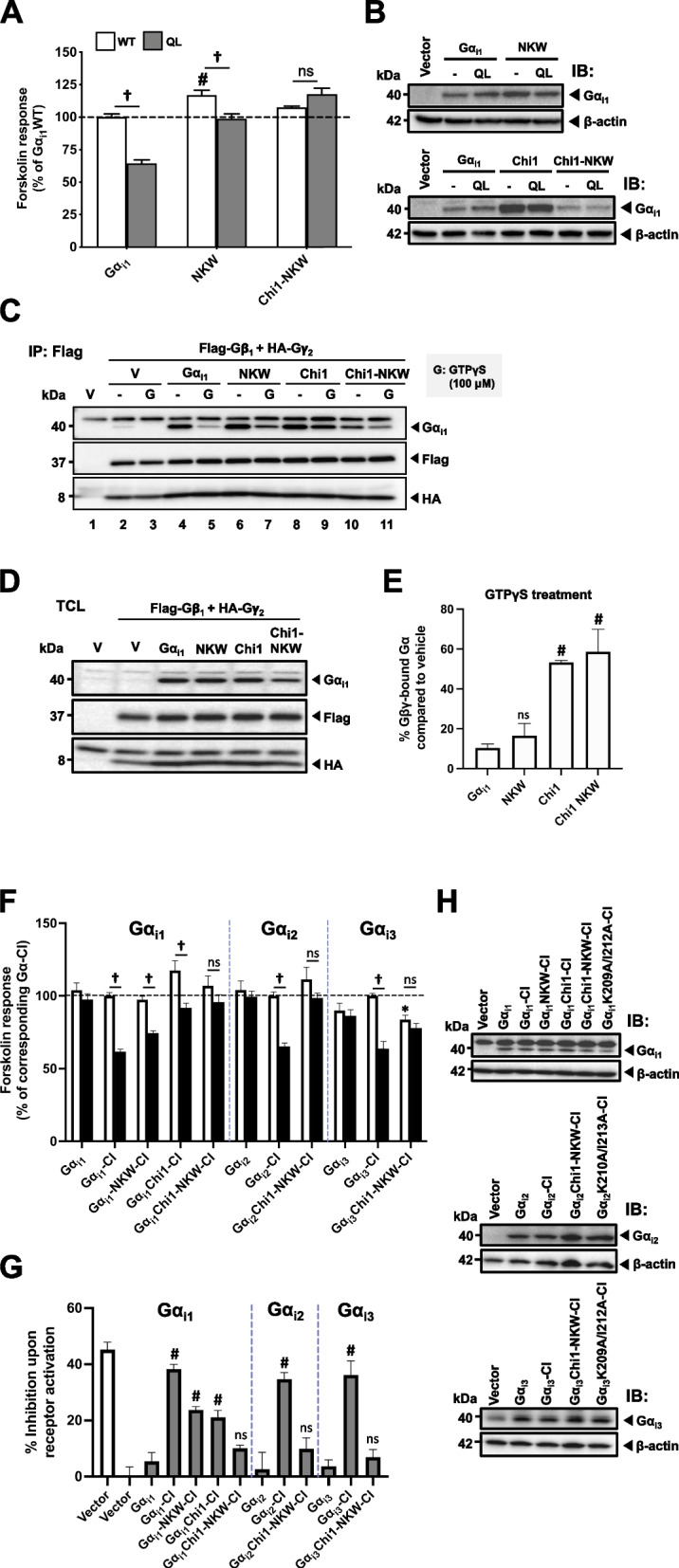
Cooperation between the α3/β5 loop, the α4 helix and the α4/β6 loop in activating AC-inhibiting Gα. For panels A and B, HEK293 cells were transfected with various QL-bearing Gα_i1_ constructs and assayed similarly to Fig. 3. For panels C to E, HEK293 cells were co-transfected with D_2_R and various Gα_i1-3_ mutants and assayed similarly to Fig. 4B. **A** The relative activities of the constitutively active chimeras are expressed as a percentage of cAMP accumulation of Gα_i1_. Data shown are mean ± SEM (*n* = 3). Bonferroni *t* test, *p* < 0.05; #, significant increase relative to control; ns, not significant; †, significant inhibition. **B**,**H** Expression of Gα_i1_ constructs were confirmed by immunoblotting with 20 μg of total protein. For panels C and D, HEK293 cells were transiently co-transfected with Flag-Gβ_1_, HA-Gγ_2_, with or without various Gα_i1_ and assayed by subunit dissociation assay as in Fig. 4D. **C** 500 μg of the total proteins of the lysate were incubated with or without 100 μM of GTPγS at 37 °C for 15 min prior to immunoprecipitation by anti-Flag affinity gel. **D** Expressions of the G proteins were confirmed by immunoblotting with 20 μg of the total proteins. **E** Quantification of the co-immunoprecipitation results. Results are expressed as a percentage of the corresponding Gα_i1_ constructs pull-down by Flag-Gβ_1_. Graph is shown as mean ± SEM (n ≥ 3). Student *t* test, *p* < 0.05; ns, non-significant; #, significantly higher than the control. **F** The forskolin-stimulated cAMP levels of the chimeras with a CI mutation are expressed as a percentage of the response normalized with the corresponding Gα-CI. **G** The forskolin response in the presence of quinpirole is expressed as a percentage inhibition of the fraction of cAMP level upon quinpirole stimulation. Data shown are mean ± SEM (*n* = 3). Bonferroni *t* test, *p* < 0.05; ns, non-significant; *, significantly lower than the control; #, significantly higher than the control; †, significant inhibition upon receptor activation

Consequently, we examined whether the replacement of N255-F259 of Gα_i1_ (NNKWF) with that of Gα_t1_ (NHRYF), named NKW (Fig. 7E), could potentially impact its ability to effectively recognize AC. NKW showed a slight activation towards AC and its QL version showed diminished constitutive activity (15.6 ± 1.6% in NKW-QL versus 35.4 ± 2.5% in Gα_i1_QL; Fig. 8A). Strikingly, the cAMP suppression contributed by the QL point mutation was abolished by a chimera combining NKW with Chi1 mutation (named Chi1-NKW-QL, -9.6 ± 4.5% inhibition) (Fig. 8A). Such observation may not be owing to the lower expression level of Chi1-NKW than Gα_i1_ and Chi1 (Fig. 8B and D), as even lesser expressions of QL/RC-bearing mutants (as seen in the case of Chi1-G183S) were adequate to inhibit AC activity (Fig. 5C and E). Moreover, NKW maintained a normal GDP/GTP exchange rate similar to the wild-type, while both Chi1 and Chi1-NKW showed a similar defect in this process (Fig. 8C and E).

We then tested if these mutants can respond to receptor activation. NKW-CI suppressed forskolin-induced cAMP elevation when D_2_R was activated, in line with our observations on other QL-bearing chimeras (Fig. 8F and G). Remarkably, Chi1-NKW-CI lost the ability to inhibit cAMP upon quinpirole treatment (Figs. 8F and G). Cognate mutations were also found to eliminate the activity of Gα_i2_ and Gα_i3_ upon receptor activation (Fig. 8F and G); the mutants were expressed in levels similar to their respective Gα (Fig. 8H). These results indicated that the α3/β5 loop, the α4 helix, and the α4/β6 loop cooperatively mediate the AC inhibition by Gα_i1-3_. They also shed light on the mechanism through which different Gα members within the same family distinguish effectors. Notably, the α3/β5 loop, the α4 helix, and the α4/β6 loop might play a pivotal role in preventing Gα_t1_ from inhibiting AC.

## Discussion

The regulation of AC activity by G proteins has long been recognized as a major signaling event which controls numerous cellular processes, but the precise mechanism remains poorly defined. Available biochemical evidence suggests that the opposing effects of Gα_s_ and Gα_i_ subunits are not due to competition for AC, since they apparently bind to different domains of the effector [49]. Recent advances in structural elucidation techniques have provided a detailed understanding on how Gα_s_ interacts with type 9 adenylyl cyclase [50]. However, far less is known pertaining to how Gα_i_ subunits inhibit AC. Given that the four AC-inhibiting Gα_i_ subunits are highly homologous, one might expect that previous experimental findings on Gα_i2_ and Gα_z_ [17–19] will be applicable to Gα_i1_, and thus allow a more precise mapping of AC-interacting residues against the Gα_i1_ crystal structures. The present study, however, suggests otherwise since substitution of putative AC-interacting domains in the chimeras failed to abolish inhibitory regulation on AC by the G_i_-coupled D_2_R (Fig. 4). We further identified a tripeptidic motif (NKW) in the α3/β5 loop as an additional region required for AC inhibition by Gα_i1-3_ (Fig. 8). This domain has been overlooked in early mapping studies, because it was assumed that the constitutively active mutants of Gα_i_, a tool commonly used in those studies [17–19], functionally mirrored that of a receptor-activated Gα_i_. Our results clearly suggested that, however, QL and RC do not resemble a receptor-activated Gα_i_. Firstly, chimeras that replaced putative AC-interacting domains of Gα_i1_ with distantly related Gα subunits could only abolish the constitutive activity arising from the RC mutation (Fig. 3). Secondly, Gα_i1_RC-CI, but not Gα_i1_QL-CI can be activated by receptors (Fig. 5A). Thirdly, RC mutation, but not QL mutation, is RGS-sensitive (Fig. 5C). Although both QL and RC mutations impede GTP hydrolysis and result in constitutive activation of the Gα subunits, it appears that Gα_i1_QL is functionally more similar to a receptor-activated Gα_i1_ in a cellular environment. As both Gln204 and Arg178 are conserved among all Gα subunits, the choice of using QL or RC mutants to demonstrate constitutive Gα activity should be carefully considered.

It is interesting to note that the present observations generally agree with previous reports [22–24], wherein the constitutive activity of the RC mutants are more prone to disruptions than the QL mutants (Fig. 3). Coleman et al. have previously compared the X-ray crystal structures of Gα_i1_ bound by GTPγS and GDP-AlF_4_^−^ respectively [42]. The slight changes in the shape of the nucleotide-binding pocket between the two crystal structures insinuated different roles of Gln204 and Arg178 in GTP hydrolysis. The glutamine residue orients a water molecule towards the γ-phosphate of GTP to initiate a nucleophilic attack, whereas the arginine residue stabilises the GDP-P_i_ transition state [42]. Thus, one may expect that the thermodynamic requirement for GTP hydrolysis could be more easily overcome in RC mutation than in QL mutation, because the latter mutation would completely abolish the initiation condition for the reaction.

The observation that Gα_i1_RC-CI suppresses AC only with receptor activation suggested that a substantial population of the molecule remains GDP-bound (Fig. 5A). This is in line with an early study on purified Gα_i1_RC and Gα_i1_QL, wherein only ~ 40% of Gα_i1_RC (as compared with 100% for Gα_i1_QL) were GTP-bound in steady-state, despite being equally GTPase-deficient [36]. Another study observed that only purified Gα_i1_RC, but not Gα_i1_QL, was sensitive to AlF_4_^−^ (a mimetic of the γ-phosphate of GTP in GDP•AlF_4_^−^-bound Gα subunits), implying that there exists a subpopulation of GDP-bound Gα_i1_RC [42]. In fact, an in vitro study on Gα_s_RC (R204C) also suggested that only around one-third of the expressed mutant was GTP-bound [55]. The conformation of purified GDP-bound Gα_s_RC resembles that of an active, GTPγS-bound Gα_s_ by stabilizing intramolecular hydrogen bonds. Furthermore, GDP-bound Gα_s_RC can bind to the catalytic domain of AC and elevate cellular cAMP level in vitro [55]. Yet, despite the strict conservation of this arginine residue in all Gα subunits, the conformation of GDP-bound Gα_i1_RC might not resemble an active Gα_i1_ at all. This postulation was supported by our observation that Gα_i1_RC-CI only exhibited inhibitory actions on AC upon receptor activation (Fig. 5A). This implies that Gα_i1_RC does not seem to stably adopt an active conformation. Alternatively, GDP-bound Gα_i1_RC may be forced to adopt an inactive conformation when it is pre-coupled to the receptor. This may explain why Gα_i1_RC constitutively inhibits AC when expressed alone, but lost its activity upon co-expression with D_2_R (Figs. 3C and 5A). The responsiveness of the RC mutants to receptor stimulation raises the possibility that active RC mutants may accumulate over time due to stimulation by endogenous receptors, which would depend on the rate of generation of GTP-bound RC mutants and their turnover rate or half-life.

Sensitivity of Gα_i1_RC towards RGS has been documented in an early reconstitution study [24]. RGS proteins are believed to replace the role of the arginine residue in stabilising the transition state during GTP hydrolysis [25]. The aid of RGS proteins is exceptionally important for members of the Gα_i/o_, such as RGS20/Gα_z_, and members of the R4 family/Gα_i1-3,oA,oB_ [56, 57]. Structural studies revealed that RGS proteins directly bind to the switch region of an activated Gα subunits and stabilise the residues for GTP hydrolysis [25, 46]. Time-resolved Fourier Transform Infrared microscopy and molecular dynamic simulation suggested that Arg-178 of Gα_i1_ interacts additionally with the α-phosphate of GTP in the presence of RGS4, hence catalysing the leaving of γ-phosphate by eclipsing all three phosphate groups of GTP, while the thermodynamic profile of Gln-204 was unaffected by RGS4 [58]. Herein, we provide the first *in cellulo* evidence that the activity of Gα_i1_RC can be turned off by RGS proteins. Chi1RC failed to suppress forskolin-mediated cAMP accumulation, but its inhibitory activity was rescued by an additional G183S mutation, which abolishes the binding of RGS proteins (Fig. 5C). This cannot be explained by potential alteration in the conformation of Gα subunit, because Chi1RC-G183S showed a similar extent of AC inhibition with Gα_i1_RC (Fig. 5C). The ability of Gα_i1_RC, but not Gα_i1_QL, to interact with RGS proteins implies that Gα_i1_RC is mainly at a GDP•P_i_ transition state of GTP hydrolysis in the cells, because RGS4 can only bind to GDP•AlF_4_^−^-complexed Gα_i1_, but not to Gα_i1_ loaded with non-hydrolysable GTPγS [59]. This further supports our postulation that a substantial fraction of cellularly expressed Gα_i1_RC is GDP-bound, which is in line with the previous reconstitution study [36]. The restoration of GTP hydrolysis of Gα_i1_RC upon RGS4 co-treatment [24], as well as our observation that Chi1RC regained AC inhibitory action with RGS uncoupling (Fig. 5C), tend to suggest a direct catalytic role of RGS proteins on the GTP hydrolysis of Gα_i1_ *in cellulo*, a function that extends beyond merely stabilising Arg-178 for the GTPase reaction as observed with RGS4 [58]. The exact molecular mechanism is open for further studies. Moreover, the Gα_i_ activity may be affected by other factors such as cellular localization and binding to other protein partners including GoLoco proteins, guanine nucleotide dissociation inhibitors, and guanine nucleotide exchange modulators.

Given that Chi1-G183S and Chi1 appeared to associate with the Gβγ dimer to similar degrees (Fig. 5G and H), the binding of Gβγ dimer and RGS proteins to Gα_i1_ may not be competitive in nature. This deviates from studies on co-crystal structures of Gα_i1_•RGS and Gα_i1_•Gβγ [25, 46, 48], wherein the two interfaces overlap. An early FRET-based study suggested that Gα_i1_ and Gβγ dimer rearrange, rather than dissociate, upon receptor activation [60]. Such structural rearrangement may be sufficient for RGS proteins to bind to an activated Gα_i1_ [61]. This is supported by the current observation that RGS proteins can bind to Chi1 despite having a significant amount of Gβγ dimer associated with the Gα subunit (Fig. 5G and H). Yet, it remains unclear if RGS proteins block AC inhibition by Chi1RC through its GAP activity, or via physical blockade of the AC-interacting surface, as seen with RGS4-inhibition of Gα_q_-mediated PLCβ1 activation by [47]. It should be noted that the coexistence of Gα_i1_•RGS and Gα_i1_•Gβγ complexes remains possible.

Based on our findings of Chi1NKW, we propose a novel mechanism of AC inhibition by the cooperation between two domains of Gα_i1-3_, including the α3/β5 loop and the region spanning the α4 helix and α4/β6 loop (Fig. 8A, F, and G). The α3/β5 loop is coplanar to the switch II region, and this plane also overlaps with the interacting surfaces of Gα_i1_ with Gβγ [48]. Moreover, a recent molecular dynamics study suggested that the C-terminal tip of Gα_i1_, which is important for receptor coupling, has strong allosteric modulation towards the Gβγ release from switch II [62]. Therefore, after receptor activation and a pipeline of structural alterations that releases the Gβγ from Gα_i1_, the exposed surface is ready for effector recognition. Notably, the α3/β5 loop likely engages in the first binding to the C1 domain of AC, while the plane of α4 helix and α4/β6 loop, which is distant from the α3/β5 loop, may provide a secondary but necessary structural refinement to elicit AC inhibition, as mutation on the α3/β5 loop alone does not abolish the inhibitory activity completely (Fig. 8A). While the Gβγ released upon receptor activation can also modulate AC activity, we believe that such influence would be minimal since the predominant AC isoforms in HEK293 cells are AC3 and AC6 [63] and they are not activated by Gβγ [64, 65]. Contemporary research on the Gα inhibitory interacting protein (GINIP) indicates that it hinders the interaction between Gα_i_ and adenylyl cyclase, thereby preventing the subsequent modulation of cAMP levels. This inhibition occurs through GINIP binding to the α3/switch II groove of active Gα_i_, which is in proximity to the α3/β5 loop [66]. Additionally, a single point mutation on W258 has been shown to disrupt the binding of GINIP to active Gα_i_ [67]. This suggests that different downstream effectors and modulators of Gα_i_ may competitively bind to this area. Further investigations are required to determine whether the α3/β5 loop region truly functions as an AC interacting site.

In summary, GTPase-deficient (and therefore constitutively active) mutants of Gα_i1_ have differential functional resemblance to a receptor-activated Gα_i1_. It is due to the distinct ability of RC to be activated by a receptor and to interact with RGS proteins. An additional structural domain, namely the α3/β5 loop, is apparently important for AC inhibition by Gα_i1-3_. Our results provide novel insights on the mechanism of AC inhibition mediated by Gα_i_, as well as deepen our understandings on the properties of two widely used switch region mutants in a cellular context.

## Materials and methods

### Materials

The cDNAs encoding various human G protein subunits and receptors were obtained from UMR cDNA Resource Center (Rolla, MO, USA). Molecular biology reagents, anti-Flag and Fluo-4 AM were purchased from Invitrogen (Carlsbad, CA, USA). Human embryonic kidney HEK293 cells (CRL-1573) were obtained from American Type Culture Collection (Rockville, MD, USA). Cell culture reagents were obtained from Thermofisher Scientific (Waltham, MA, USA). Polyethylenimine (PEI) (Linear, MW 25,000) was purchased from Polysciences, Inc. (Warrington, PA, USA). Pertussis toxin (PTX) was ordered from List Biological Laboratories (Campbell, CA, USA). Forskolin and quinpirole hydrochloride were purchased from Tocris Bioscience (Bristol, UK). The [^3^H]adenine was purchased from American Radiolabeled Chemicals (St. Louis, MO, USA) and PerkinElmer (Waltham, MA, USA), while the scintillation fluid (Optiphase Hisafe 3) and [^3^H]inositol were purchased from PerkinElmer (Waltham, MA, USA). Anti-Gα_i1_ primary antibody was from Aviva Systems Biology (San Diego, CA, USA). Anti-Gα_i2_ and anti-Gα_i3_ antibodies were from Santa Cruz Biotechnology (Santa Cruz, CA, USA). Anti-HA and anti-β-actin were from Roche Molecular Biochemicals (Indianapolis, IN, USA). EZview™ Red ANTI-FLAG® M2 Affinity Gel and other chemicals were purchased from Sigma (St. Louis, MO, USA).

### Construction of Gα_i_ mutants

The respective DNA fragments were amplified by polymerase chain reaction (PCR), with the reaction mixture and thermal cycle conditions in accordance with the manufacturer’s protocol. Full-length, mutated Gα_i1_ cDNA was constructed by overlapping PCR. Primers used for the amplification of fragments are provided in Table 4. The cDNA was cloned into *HindIII* and *XbaI* sites of the pcDNA3.1( +) vector by standard restriction digestion and T4 ligation. Each construct generated was confirmed by Sanger sequencing.

**Table 4:** List of primers

Construct	Junction/Mutation	Primer sequence (5′ 3’)
Chi1	Gα_i1_ Gα_t1_ (297–318)	**F:** ATATGTGTTTGATCCTGCATATTCTGG
		**R:** CCAGAATATGCAGGATCAAACACATATGAGGACGCCGGCAACTAC
		**F:** GGCACATGTGAAGTGGGTGTATATCTCCTTCACGTCGCGCC
		**R:** ATATACACCCACTTCACATGTGCC
Chi2 & Chi4	Gα_i1_ Gα_t1_ (228–236)	**F:** AGCGCCTACGACATGGTGCTAGTGGAGGATGAAGAAATGAACCGAATGC
		**R:** CTCCACTAGCACCATGTCGTAGGCGCTCAGTGCTACACAGAAGATGATCGCCG
Chi3	Gα_i1_ Gα_t1_ (297–355)	**F:** CCAGAATATGCAGGATCAAACACATATGAGGACGCCGGCAACTAC
		**R:** TAGAAGGCACAGTCGAGG
Chi5	Gα_i1_ Gα_q_ (297–318)	**F:** ATATGTGTTTGATCCTGCATATTCTGG
		**R:** CCAGAATATGCAGGATCAAACACATATGCCCAGGCAGCCCGAGAATTC
		**F:** GGCACATGTGAAGTGGGTGTATATAATTTTGTCACTGTCTGG
		**R:** ATATACACCCACTTCACATGTGCC
Chi6	Gα_i1_ Gα_q_ (297–349)	**F:** CCAGAATATGCAGGATCAAACACATATGCCCAGGCAGCCCGAGAATTC
		**R:** CCCTCTAGATTAAAAGAGACCACAATCCTTCAGGTTCAACTGGAGGATGG
Chi1-KH	Gα_i1_K248H	**F:** GCATGAAAGCATGCACTTGTTTGACAGC
		**R:** GCTGTCAAACAAGTGCATGCTTTCATGC
Chi1-DN	Gα_i1_D251N	**F:** CATGAAATTGTTTAACAGCATATGTAAC
		**R:** GTTACATATGCTGTTAAACAATTTCATG
Chi1-KH/DN	Gα_i1_K248H/D251N	**F:** GCATGAAAGCATGCACTTGTTTAACAGCATATGTAAC
		**R:** GTTACATATGCTGTTAAACAAGTGCATGCTTTCATGC
Chi1-KDY, Chi2-KDY, Chi3-KDY	Gα_i1_Y287F	**F:** CACTATATGCTTTCCAGAATATGC
		**R:** GCATATTCTGGAAAGCATATAGTG
Chi1-AAA	Gα_i1_E238A/E239A/M240A	**F:** CTAGCTGAAGATGCAGCAGCAAACCGAATGC
		**R:** GCATTCGGTTTGCTGCTGCATCTTCAGCTAG
Gα_i1_-G183S & Chi1-G183S	Gα_i1_G183S	**F:** GTGAAAACTACAAGCATTGTTGAAACC
		**R:** GGTTTCAACAATGCTTGTAGTTTTCAC
Gα_i1_-I212A	Gα_i1_I212A	**F:** CGGAAGAAGTGGGCTCATTGCTTC
		**R:** GAAGCAATGAGCCCACTTCTTCCG
Gα_i1_-I212L	Gα_i1_I212L	**F:** CGGAAGAAGTGGCTTCATTGCTTC
		**R:** GAAGCAATGAAGCCACTTCTTCCG
Gα_i1_-I212V	Gα_i1_I212V	**F:** CGGAAGAAGTGGGTTCATTGCTTC
		**R:** GAAGCAATGAACCCACTTCTTCCG
Gα_i1_-I212F	Gα_i1_I212F	**F:** CGGAAGAAGTGGTTTCATTGCTTC
		**R:** GAAGCAATGAAACCACTTCTTCCG
Gα_i1_-K209A/I212A	Gα_i1_K209A	**F:** GATCTGAGCGGGCGAAGTGGGCTC
		**R:** GAGCCCACTTCGCCCGCTCAGATC
Gα_i1_-NKW or Chi1-NKW	Gα_i1_N256H/K257R/W258Y	**F:** GCATATGTAACCACCGCTACTTTACAGATAC
		**R:** GTATCTGTAAAGTAGCGGTGGTTACATATGC
Gα_i1_-K35A	Gα_i1_K35A	**F:** CGCGAGGTCGCACTGCTGCTG
		**R:** CAGCAGCAGTGCGACCTCGCG
Gα_i1_-H188A	Gα_i1_H188A	**F:** GTTGAAACCGCATTTACTTTC
		**R:** GAAAGTAAATGCGGTTTCAAC
Gα_i1_-K197A	Gα_i1_K197A	**F:** CTTCATTTTGCAATGTTTGAT
		**R:** ATCAAACATTGCAAAATGAAG
Gα_i1_-E216A K257A	Gα_i1_E216A Gα_i1_K257A	**F:** CATTGCTTCGCAGGAGTGACG
		**R:** CGTCACTCCTGCGAAGCAATG **F:** GTAACAACGCGTGGTTTACAG **R:** CTGTAAACCACGCGTTGTTAC
Gα_i1_-E216K K257E	Gα_i1_E216K Gα_i1_K257E	**F:** CATTGCTTCAAAGGAGTGACG
		**R:** CGTCACTCCTTTGAAGCAATG **F:** GTAACAACGAGTGGTTTACAG **R:** CTGTAAACCACTCGTTGTTAC
Gα_i1_-PLT	Gα_i1_P282H/T284S	**F:** GCATATGTAACCACCGCTACTTTACAGATAC **R:** GTATCTGTAAAGTAGCGGTGGTTACATATGC
Gα_i2_-K210A/I213A	Gα_i2_K210A/I213A	**F:** GTCTGAGCGGGCAAAGTGGGCACACTGCTTTG
		**R:** CAAAGCAGTGTGCCCACTTTGCCCGCTCAGAC
Gα_i2_-Chi1-NKW	Gα_i2_ Gα_t1_ (298–319)	**F:** GCCAACAAATATGAGGACGCCGGCAACTAC
		**R:** GCCGGCGTCCTCATATTTGTTGGCCCCTGTGTAC
		**F:** GACGTGAAGGAGATCTACACGCACTTCACG
		**R:** GTGCGTGTAGATCTCCTTCACGTCGCGCCGCATG
	Gα_i2_N257H/K258R/W259Y	**F:** CATCTGCAACCACCGCTACTTCACAGAC
		**R:** GTCTGTGAAGTAGCGGTGGTTGCAGATG
Gα_i3_-CI	Gα_i3_C351I	**F:** TAATACGACTCACTATAGGG
		**R:** GCCCTCTAGACTCGAGTCAATAAAGTCCGATTTCCTTTAAG
Gα_i3_-K209A/I212A-CI	Gα_i3_K209A/I212A	**F:** GATCAGAACGAGCAAAGTGGGCACACTGTTTTG
		**R:** CAAAACAGTGTGCCCACTTTGCTCGTTCTGATC
Gα_i3_-Chi1-NKW	Gα_i3_ Gα_t1_ (297–318)	**F:** TCCAATACATATGAGGACGCCGGCAACTAC
		**R:** GCCGGCGTCCTCATATGTATTGGAACCTGTGTATTC
		**F:** GACGTGAAGGAGATCTATACTCACTTCACC
		**R:** GTGAGTATAGATCTCCTTCACGTCGCGCCGCATG
	Gα_i3_N256H/K257R/W258Y	**F:** CATTTGTAATCACCGCTACTTTACAGAAAC
		**R:** GTTTCTGTAAAGTAGCGGTGATTACAAATG

Except Gα_i2_-CI and Gα_i3_-CI, all mutations to Gα cDNA were made by overlapping PCR using the above primers and the outermost primers (Forward (F): T7 [TAATACGACTCACTATAGGG]; Reverse (R): BGH [TAGAAGGCACAGTCGAGG]), using a template of Gα bearing QL/RC/CI wherever necessary. Gα_i2_-CI and Gα_i3_-CI were created by direct PCR on Gα_i2_ and Gα_i3_ cDNA, respectively

### Transient transfection

HEK293 cells were maintained in MEM supplemented with 10% (v/v) of FBS (MEM/FBS), 100 units/mL penicillin and 100 μg/mL streptomycin, incubated at 37 °C in a humidified atmosphere with 5% CO_2_. Cells were transiently transfected by PEI transfection system. Briefly, DNA-PEI mixture was prepared by mixing 0.4 μg of plasmid DNA with 50 μL of 150 mM NaCl and 1.6 μL of PEI solution (1 mg/mL). The mixture was vortexed for 10 s and incubated at room temperature for 15 min. Cells in 12-well plates were also fed with 700 μL of fresh MEM/FBS. After 15 min of incubation, 50 μL of DNA-PEI mixture was transferred into each well and was gently mixed. Cells were assayed two days after transfection.

### cAMP accumulation assay

Transfected cells were labeled with 1 μCi/mL of [^3^H]adenine in MEM with 10% (v/v) FBS and treated with 100 ng/mL PTX as appropriate one day after transfection. Labeled cells were challenged in serum-free media with 50 μM forskolin and 1 mM 1-methyl-3-isobutylxanthine, in the absence or presence of 100 nM quinpirole for 30 min. Treatments were terminated by 1 mL of ice-cold stop solution containing 5% (w/v) trichloroacetic acid with 1 mM ATP. Separation of tritiated cAMP from other adenosines was performed by sequential ion exchange chromatography as described previously [19]. The ratios of [^3^H]cAMP to total [^3^H]ATP, [^3^H]ADP, and [^3^H]cAMP pools were determined. To facilitate comparisons of the inhibitory responses of various chimeras across different experiments, results were expressed as a percentage of forskolin response obtained with the corresponding control, Gα_i1_. Absolute values for cAMP accumulation varied between experiments, but cAMP/Total (× 1000) values for forskolin-induced responses typically ranged from 80–120; variability within a given experiment was < 10% in general.

### Inositol phosphates (IP) accumulation assay

Transfected cells were labeled with 2 μCi/mL of *myo*-[^3^H]inositol in 10% (v/v) FBS-containing MEM and treated with PTX whenever necessary. Labeled cells were treated with or without 100 nM quinpirole in serum-free media containing 20 mM LiCl for 1 h, and the reaction was stopped by 0.75 mL of 20 mM formic acid. [^3^H]IP produced was separated from the total [^3^H]inositol pool by sequential ion exchange chromatography similarly to previous literature [68]. For Ca^2+^ assay, transfectants were transferred into 96-well clear bottom plates and then treated with 100 ng/mL PTX overnight where appropriate. Culture media were then removed followed by cell labeling with 2 μM Fluo-4 AM in HBSS supplemented with 20 mM HEPES (pH 7.5) and 2.5 mM probenecid for 45 min at 37 °C. After the labeling, cells were treated with various doses of quinpirole. Changes in fluorescence were monitored by the FLIPR system with the excitation wavelength of 488 nm as previously described [69].

### Western blotting analysis

Transfected cells were lysed by SDS-containing sample buffer (60 mM Tris–HCl (pH 6.8), 5% (v/v) glycerol, 1.7% (w/v) SDS, 1.6% (w/v) dithiothreitol, bromophenol blue). Proteins were separated by 12% SDS–polyacrylamide gel and transferred to nitrocellulose membrane. The membrane was incubated with required antibodies, and chemiluminescence was recorded by the ChemiDoc Touch Imaging System (BioRad, Hercules, CA, USA). Quantification of protein band intensities was performed in ImageJ.

### Molecular modeling and sequence alignment

Crystallographic structures were downloaded from the RCSB Protein Data Bank (National Institute of Health, Bethesda, MD, USA), with the PDB codes stated in the figure legends. Alignment of the amino acid sequences of Gα subunit was done by the Clustal Omega multiple sequence alignment program (EMBL-EDI, Hinxton, UK). Analyses on the three-dimensional structures of proteins were performed on PyMOL 2.4.

### Molecular Docking of Gα_i1_-AC5/6 models using HADDOCK 2.4

To model the interactions between Gα_i1_ and AC, the High Ambiguity Driven protein–protein DOCKing software (HADDOCK 2.4) was used, which is accessible at http://wenmr.science.uu.nl/haddock2.4/. The input structures encompassed the AlphaFold-generated structures of human adenylyl cyclase 5 and 6 (Uniprot ID: O95622-1 and O43306-1), along with the X-ray crystallographic structure of Gα_i1_ (PDB: 1GFI). The docking process was facilitated through the GURU interface provided by HADDOCK, which requires the inputs of ambiguous interaction restraints (AIRs). AIRs were defined at the binding interface, categorized as "active" and "passive" residues. The "active" residues, informed by experimental data, were directly implicated in the interaction, while the adjacent residues, designated as "passive," were included to account for their close proximity. Subsequent to docking simulations, the model clusters with the lowest HADDOCK scores were further analyzed using PRODIGY and the representative model was visualized using PyMOL 2.4.

### Statistical analysis

The cAMP (or IP) levels were calculated as 1000 × [the ratios of the count-per-min of specific fractions ([^3^H]cAMP or [^3^H]IP) to those of total fractions ([^3^H]adenosine phosphates or [^3^H]inositol). Data shown in the figures were the mean ± SEM of three independent experiments performed in triplicates. The data sets were analyzed by ANOVA and Bonferroni *t* test with 95% confidence. The relative fluorescent unit (RFU) in the Ca^2+^ FLIPR™ assay was calculated by the background-subtracted difference between the maximum and the minimum fluorescence throughout the time course. The corresponding data was expressed as mean ± SD. Data from subunit dissociation assay were represented as mean ± SEM of three individual experiments and was analyzed by Student *t* test. All statistical analyses were performed by GraphPad Prism 8.

### Supplementary Information

**Additional file 1:** **Fig. S1.** Signaling by Gα_i1/q_ chimeras. (A) HEK293 cells were transfected with 0.4 μg/mL of various Gα_i1/t1_ constructs. Cells were labeled with *myo*-[^3^H]inositol and then assayed for [^3^H]IP accumulation. Data shown are mean ± SEM of one triplicate experiment. (B) Expression of Gα_i1_ or Gα_q_ constructs were confirmed by immunoblotting with 20 μg of total protein.

**Additional file 2:** **Fig. S2.** G_q_-signaling by Chi5-CI upon receptor activation. (A) The D_2_R-induced IP accumulations of Gα_i1_ or Gα_q_ constructs. HEK293 cells were co-transfected with D_2_R and various Gα_i1_ or Gα_q_ constructs (0.2 μg/mL each), followed by an overnight labeling with *myo*-[^3^H]inositol and pretreatment with PTX (100 ng/mL) one day after transfection. Cells were assayed for [^3^H]IP production as in Fig. 4C. Data shown are mean ± SEM of a representative experiment. (B) FLIPR assay on intracellular calcium level upon D_2_R stimulation. Transfected cells were labeled with Fluo-4 AM for 45 min, followed by 2-min detection of fluorescence immediately after the application of different concentrations of quinpirole. Data shown are mean ± SEM (*n*=3).

**Additional file 3: Fig. S3.** Preserved inhibitory function of Gα_i1_ in other investigated sites. HEK293 cells were transfected with QL-bearing Gα_i1_constructs and assayed as in Fig. 3B. The relative activities of the constitutively active mutants are expressed as a percentage of cAMP accumulation of Gα_i1_. Data shown are mean ± SEM (*n*=3). Bonferroni *t* test, *p* < 0.05; †, significant inhibition; #, significantly higher than the control; *, significantly lower than the control.

**Additional file 4:** **Fig. S4.** PDBsum prediction on electrostatic interactions on the Gα_i1_-AC interface. Predicted hydrogen bonds (in blue solid line), salt bridges (in red solid line), and non-bonded contacts (in orange dashed line) formed between residues of Gα_i1_ and (A) AC5 or (B) AC6 are indicated. The filled color represents residue categorization, with blue indicating positive residues (H, K, R), red indicating negative residues (D, E), green indicating neutral residues (S, T, N, Q), gray indicating aliphatic residues (A, V, L, I, M), purple indicating aromatic residues (F, Y, W), brown indicating Proline and Glycine (P, G), and yellow indicating Cysteine (C). Residues subject to mutations are marked with asterisk (*). By comparing (A) and (B), there are several shared interactions between Gα_i1_-AC5 and Gα_i1_-AC6, which include K209-C485/395, K210-E489/399, H213-M492/402, R208-T555/465, I212-T493/403, K257-L550/460, S252-E553/463, and F215-V554/464.

## Data Availability

All data included in this study, information on the materials and methods are available from the corresponding author upon request.

## Abbreviations

AC: Adenylyl cyclase
cAMP: 3’,5’-Cyclic adenosine monophosphate
D_2_R: Dopamine D_2_ receptor
GAP: GTPase-activating protein
GINIP: Gα inhibitory interacting protein
GPCR: G protein-coupled receptor
GTPγS: Guanosine 5’-O-[γ-thio]-triphosphate
IP: Inositol phosphates
PDEγ: Phosphodiesterase γ
PLCβ: Phospholipase Cβ
PTX: Pertussis toxin
RGS: Regulator of G protein signaling
WT: Wildtype
