# Supplementary figures and images for: Inhibition of adenylyl cyclase by GTPase-deficient Gαi is mechanistically different from that mediated by receptor-activated Gαi
Source: Cell Commun Signal. 2024 Apr 5;22:218. doi: 10.1186/s12964-024-01572-3 (PMC10996109; doi:10.1186/s12964-024-01572-3)

## Slide 1
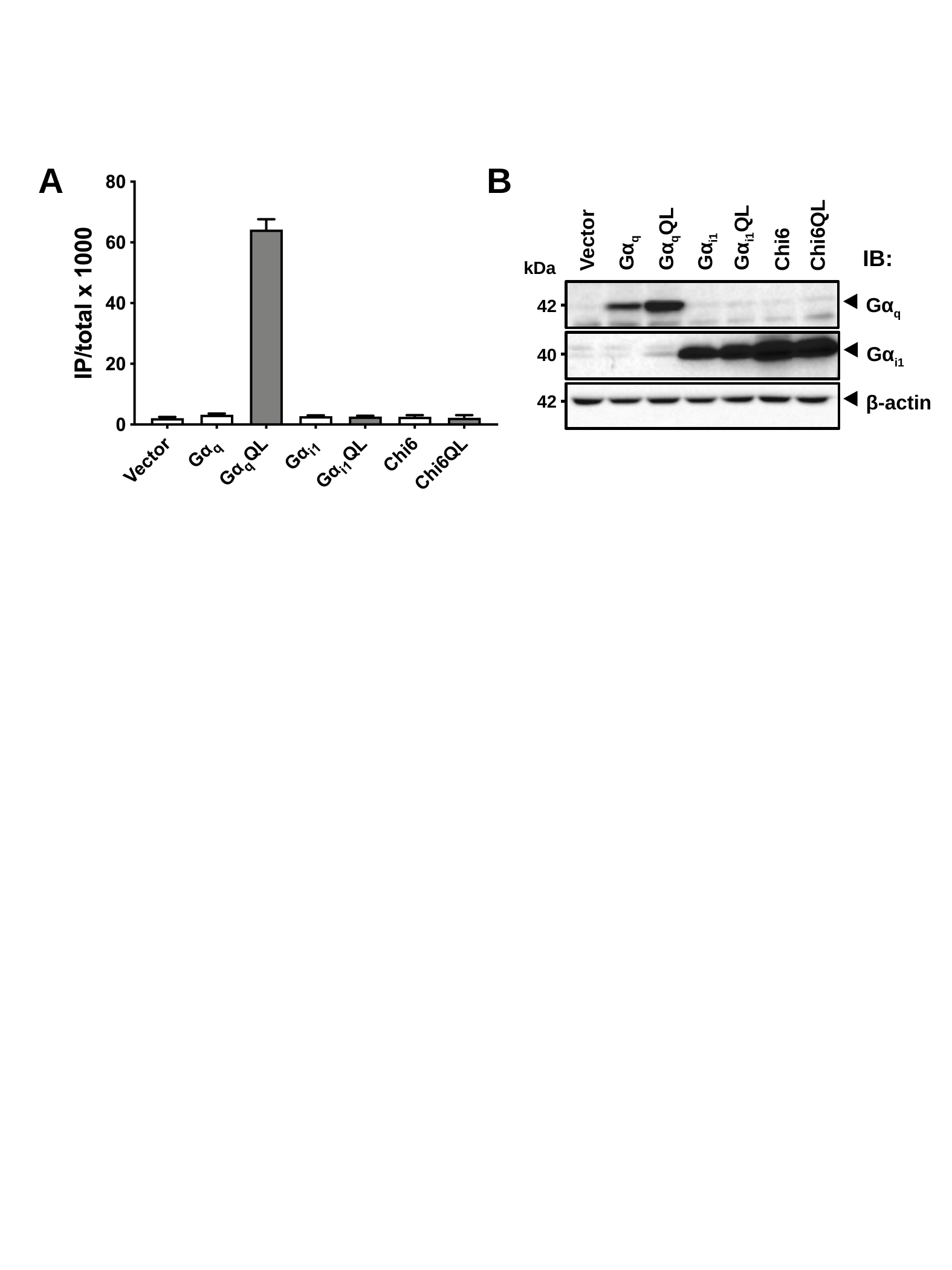

A
B
Chi6QL
Gαi1QL
GαqQL
Vector
Chi6
Gαi1
Gαq
IB:
kDa
Gαq
42
Gαi1
40
β-actin
42

Supplement: Supplementary file 1 — Additional file 1: Fig. S1. Signaling by Gαi1/q chimeras. (A) HEK293 cells were transfected with 0.4 μg/mL of various Gαi1/t1 constructs. Cells were labeled with myo-[3H]inositol and then assayed for [3H]IP accumulation. Data shown are mean ± SEM of one triplicate experiment. (B) Expression of Gαi1 or Gαq constructs were confirmed by immunoblotting with 20 μg of total protein. [file 12964_2024_1572_MOESM1_ESM.pptx]

## Slide 1
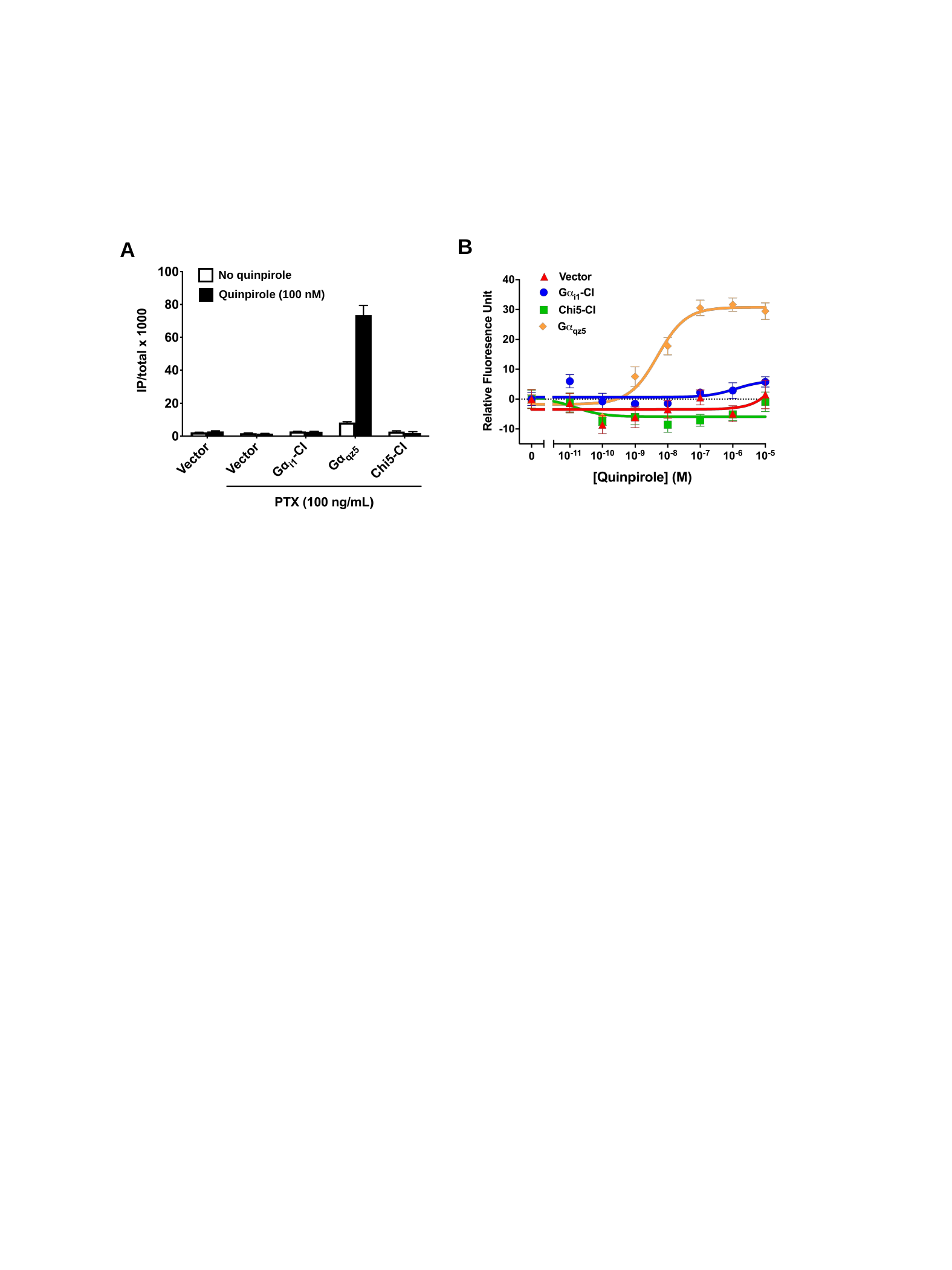

B
A
No quinpirole
Quinpirole (100 nM)

Supplement: Supplementary file 2 — Additional file 2: Fig. S2. Gq-signaling by Chi5-CI upon receptor activation. (A) The D2R-induced IP accumulations of Gαi1 or Gαq constructs. HEK293 cells were co-transfected with D2R and various Gαi1 or Gαq constructs (0.2 μg/mL each), followed by an overnight labeling with myo-[3H]inositol and pretreatment with PTX (100 ng/mL) one day after transfection. Cells were assayed for [3H]IP production as in Fig. 4C. Data shown are mean ± SEM of a representative experiment. (B) FLIPR assay on intracellular calcium level upon D2R stimulation. Transfected cells were labeled with Fluo-4 AM for 45 min, followed by 2-min detection of fluorescence immediately after the application of different concentrations of quinpirole. Data shown are mean ± SEM (n=3). [file 12964_2024_1572_MOESM2_ESM.pptx]

## Slide 1
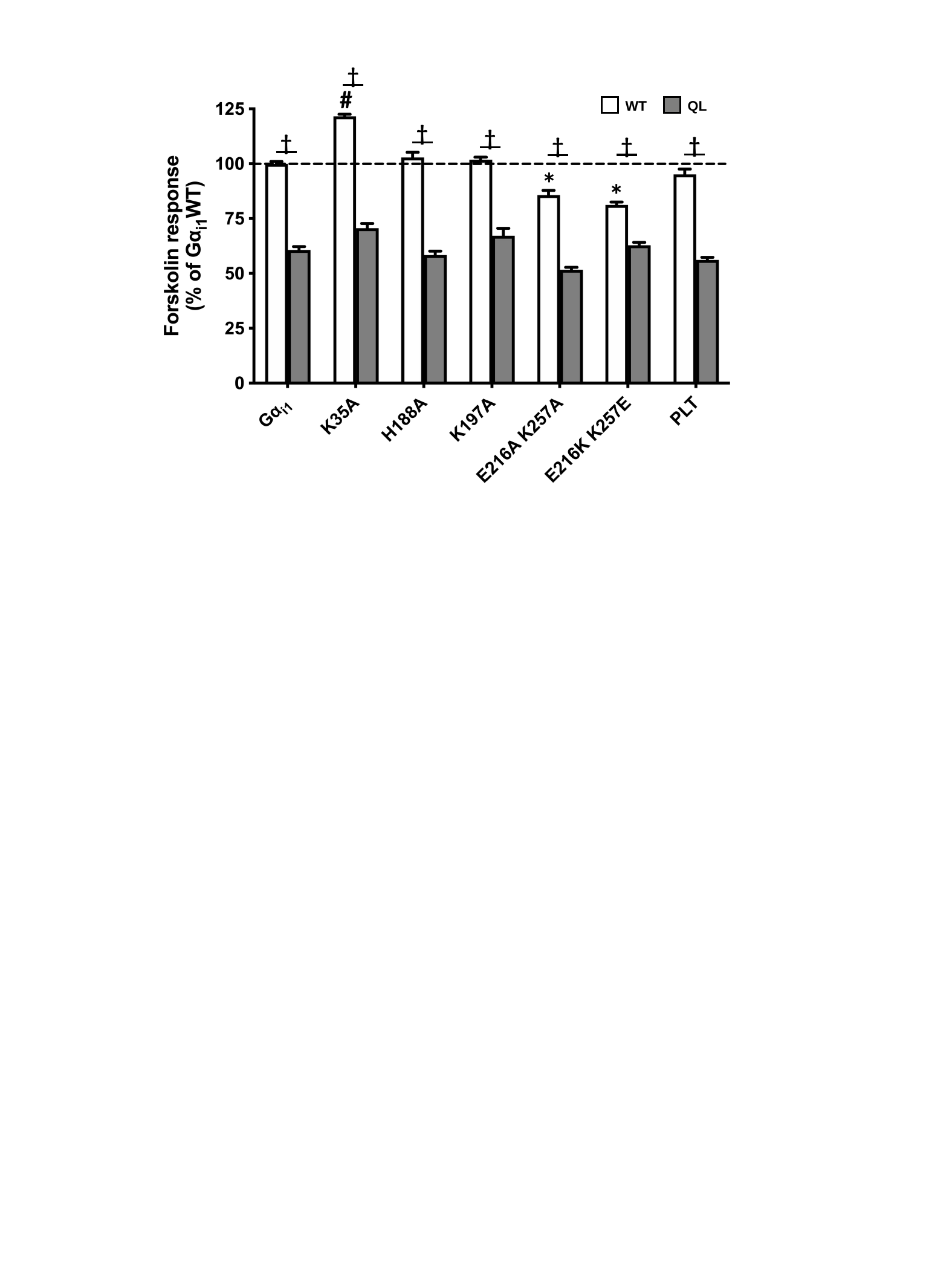

†
#
WT
QL
†
†
†
†
†
†
*
*

Supplement: Supplementary file 3 — Additional file 3: Fig. S3. Preserved inhibitory function of Gαi1 in other investigated sites. HEK293 cells were transfected with QL-bearing Gαi1constructs and assayed as in Fig. 3B. The relative activities of the constitutively active mutants are expressed as a percentage of cAMP accumulation of Gαi1. Data shown are mean ± SEM (n=3). Bonferroni t test, p < 0.05; †, significant inhibition; #, significantly higher than the control; *, significantly lower than the control. [file 12964_2024_1572_MOESM3_ESM.pptx]
