# Supplementary material for: Inhibition of adenylyl cyclase by GTPase-deficient Gαi is mechanistically different from that mediated by receptor-activated Gαi
Source: Cell Commun Signal. 2024 Apr 5;22:218. doi: 10.1186/s12964-024-01572-3 (PMC10996109; doi:10.1186/s12964-024-01572-3)
Supplement: Supplementary file 4 — Additional file 4: Fig. S4. PDBsum prediction on electrostatic interactions on the Gαi1-AC interface. Predicted hydrogen bonds (in blue solid line), salt bridges (in red solid line), and non-bonded contacts (in orange dashed line) formed between residues of Gαi1 and (A) AC5 or (B) AC6 are indicated. The filled color represents residue categorization, with blue indicating positive residues (H, K, R), red indicating negative residues (D, E), green indicating neutral residues (S, T, N, Q), gray indicating aliphatic residues (A, V, L, I, M), purple indicating aromatic residues (F, Y, W), brown indicating Proline and Glycine (P, G), and yellow indicating Cysteine (C). Residues subject to mutations are marked with asterisk (*). By comparing (A) and (B), there are several shared interactions between Gαi1-AC5 and Gαi1-AC6, which include K209-C485/395, K210-E489/399, H213-M492/402, R208-T555/465, I212-T493/403, K257-L550/460, S252-E553/463, and F215-V554/464. [file 12964_2024_1572_MOESM4_ESM.pptx]

## Slide 1
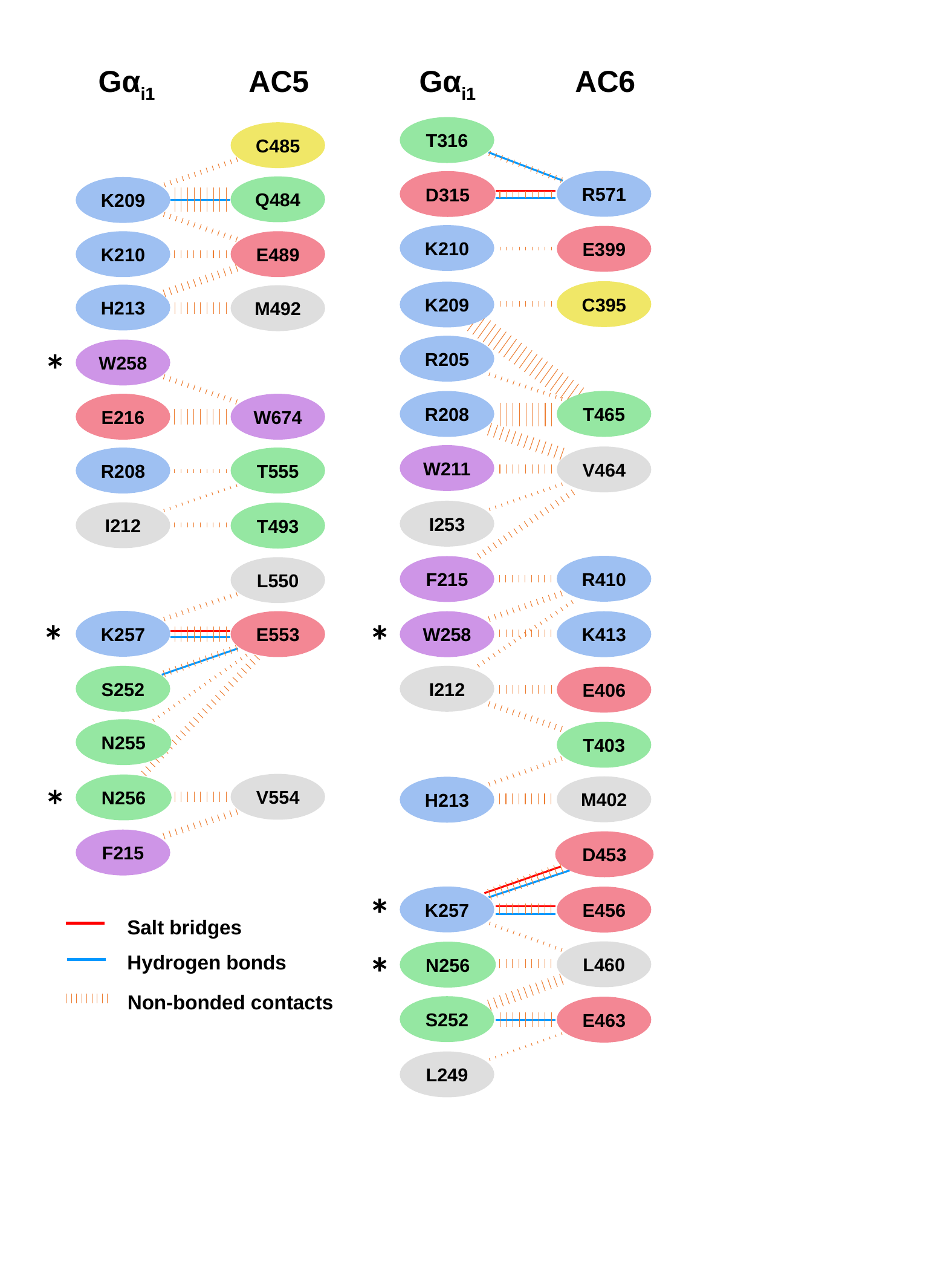

Gαi1
AC5
Gαi1
AC6
T316
C485
R571
D315
Q484
K209
K210
E399
E489
K210
C395
K209
H213
M492
R205
*
W258
T465
R208
E216
W674
W211
V464
R208
T555
I253
I212
T493
R410
F215
L550
*
*
K257
E553
W258
K413
S252
I212
E406
N255
T403
*
V554
N256
M402
H213
F215
D453
*
K257
E456
Salt bridges
*
L460
N256
Hydrogen bonds
Non-bonded contacts
S252
E463
L249
